# Augmented 3D Printing for Multiscale Microphysiological Systems

**DOI:** 10.1002/smll.202504750

**Published:** 2025-07-09

**Authors:** Kyeong Seob Hwang, Jiyoung Song, Hyun Wook Kang, Jongbaeg Kim, Cheol Woo Ha, Nakwon Choi, Seokyoung Bang, Hong Nam Kim

**Affiliations:** ^1^ Brain Science Institute Korea Institute of Science and Technology (KIST) Seoul 02792 Republic of Korea; ^2^ School of Mechanical Engineering Yonsei University Seoul 03722 Republic of Korea; ^3^ School of Mechanical Engineering Korea University Seoul 02841 Republic of Korea; ^4^ Korea Additive Manufacturing Innovation Center Korea Institute of Industrial Technology Siheung‐si 15014 Republic of Korea; ^5^ Department of Biomedical Sciences College of Medicine Korea University Seoul 02841 Republic of Korea; ^6^ Department of Convergence Medicine College of Medicine Korea University Seoul 02841 Republic of Korea; ^7^ Institute of Human Genetics College of Medicine Korea University Seoul 02841 Republic of Korea; ^8^ Research Institute for Convergence Biomedical Science College of Medicine Korea University Seoul 02841 Republic of Korea; ^9^ Department of Biomedical Engineering Dongguk University Goyang 10326 Republic of Korea; ^10^ Division of Bio‐Medical Science & Technology KIST School Korea University of Science and Technology Seoul 02792 Republic of Korea; ^11^ Yonsei‐KIST Convergence Research Institute Yonsei University Seoul 03722 Republic of Korea

**Keywords:** 3D printing, augmented 3D printing, microphysiological system, multiscale ancillary techniques

## Abstract

Microphysiological systems (MPS) can recapitulate physiological features of human organs; however, existing engineering techniques are limited to fabricating sophisticated and functional MPS. Although 3D printing offers the potential to enhance the complexity of MPS and simplify the fabrication process, existing 3D‐printed MPS suffer from challenges in fabricating multiscale structures spanning nanometers to centimeters. Recent studies suggested that these issues can be addressed by integrating multiscale ancillary techniques into 3D printing. In this review, an overview of augmented 3D‐printing techniques is provided, combined with multiscale ancillary techniques for multiscale MPS fabrication, which are termed augmented 3D‐printing techniques. It starts by providing an overview of 3D‐printing methods and relevant multiscale ancillary techniques. Then, recent developments are highlighted in augmented 3D‐printed MPS that show features that surpass those of conventional systems. This augmented approach opens the door to MPS with more physiologically relevant structures and functions, thereby marking a significant step forward in next‐generation tissue engineering.

## Graphical abstract

This review explores augmented 3D printing techniques combined with multiscale ancillary methods to overcome current limitations in fabricating complex microphysiological systems (MPS). It covers 3D printing and ancillary techniques, and highlights advancements in augmented MPS that surpass conventional systems, offering more physiologically relevant structures and functions for next‐generation tissue engineering.

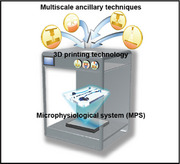

## 1 Introduction

Microphysiological systems (MPSs), which are referred to as organ‐on‐a‐chip, are in vitro models designed to replicate key aspects of human biology, including tissue structure, cellular organization, and physiological responses.^[^ ^1^ ^]^ These platforms integrate in vitro cell culture, microfabrication, and microfluidics to recapitulate the cellular microenvironment.^[^ ^2^ ^]^ Conventional in vitro cell cultures involve 2D, rigid, and flat surfaces that differ significantly from complex conditions found in living tissues.^[^ ^3^ ^]^ MPS has been proposed as an alternative platform for overcoming these limitations because it enables precise control over the microenvironment at a cellular level, including spatiotemporal cell distribution, biochemical gradient generation, and reconstitution of tissue‐like mechanical properties.^[^ ^4^ ^]^ This advanced microenvironment engineering enables MPS to replicate the physiological features of living tissues within an engineered system.^[^ ^5^ ^]^ This capability significantly enhances their biomedical relevance in applications such as drug screening and disease modeling.^[^ ^6^ ^]^

As a physical compartment of the MPS, a scaffold (e.g., a hydrogel) serves as a niche for cells, whereas microstructures guide scaffold patterning. Consequently, the engineering of the MPS microenvironment is determined by the design of these microstructures. For example, soft tissues such as adipose or brain tissues can be reconstituted by sustaining their structures through a microfabricated MPS.^[^ ^7^ ^]^ Since its emergence, MPSs have been fabricated by replicating predefined microstructures in molds using polydimethylsiloxane (PDMS), which is a transparent rubber that solidifies after heat treatment.^[^ ^8^ ^]^ These processes require mold preparation, which has several limitations, including long preparation times and difficulty in design modification. Recently, the success of additive manufacturing has paved the way for using 3D printing technology to fabricate MPS chips. A wide range of MPS chips has been developed for effectively addressing limitations associated with traditional MPS fabrication methods.

Meanwhile, 3D bioprinting techniques have been introduced, which enable the simultaneous printing of multiple cell‐hydrogel mixtures. However, cell‐laden natural hydrogels often exhibit weak mechanical properties, which makes it challenging to maintain their structures after printing.^[^ ^9^ ^]^ To address this issue, support materials that stabilize the structure or housing frames of bioprinted hydrogels are printed simultaneously.^[^ ^10^ ^]^ The use of synthetic hydrogels for bioprinting has been explored to overcome the poor mechanical properties of natural hydrogels.^[^ ^11^ ^]^ However, synthetic hydrogels often lack bioactive ligands and can produce toxic degradation products, which results in bioprinted parts with inferior biocompatibility compared to those made with natural hydrogels.^[^ ^12^ ^]^ In addition, in microfluidic environments, synthetic hydrogels reduce the angiogenic and vasculogenic capabilities of endothelial cells, thereby posing challenges to microvascularization in tissues.^[^ ^13^ ^]^ Further, 3D bioprinting has a low resolution, which limits its ability to accurately replicate tissue ultrastructures.^[^ ^14^ ^]^

Compared with conventional MPS chips fabricated using photolithography and soft lithography, 3D‐printed MPS chips offer several advantages: 1) Design modifications can be implemented quickly and easily.^[^ ^15^ ^]^ Unlike conventional soft lithography methods, which require multiple steps such as photolithography, surface modification, replication, and plasma bonding, design changes in 3D printing can be instantly applied once finalized. 2) 3D printing enables the fabricating of complex structures with significant heights. In photolithography‐based mold preparation using SU‐8 as a photoresist, the microchannel height is limited to a few hundred micrometers. In addition, the high viscosity of SU‐8 can lead to an uneven thickness during spin coating.^[^ ^16^ ^]^ Processes involving baking, alignment, exposure, and development must be repeated multiple times to produce complex shapes using photolithography, which makes them time‐consuming and labor‐intensive.^[^ ^15b^ ^]^ In contrast, 3D printing offers a simplified manufacturing process regardless of the complexity or size of the structure.^[^ ^17^ ^]^ 3) 3D printing is well‐suited for prototyping MPS chips before transitioning to mass production. Recent studies have used 3D‐printing technology to optimize MPS designs for roll‐to‐roll production processes.^[^ ^18^ ^]^ In addition, several studies explored the use of 3D‐printing techniques to refine MPS chip designs, which can later be adapted for injection‐molded production.^[^ ^19^ ^]^

**Figure 1:**
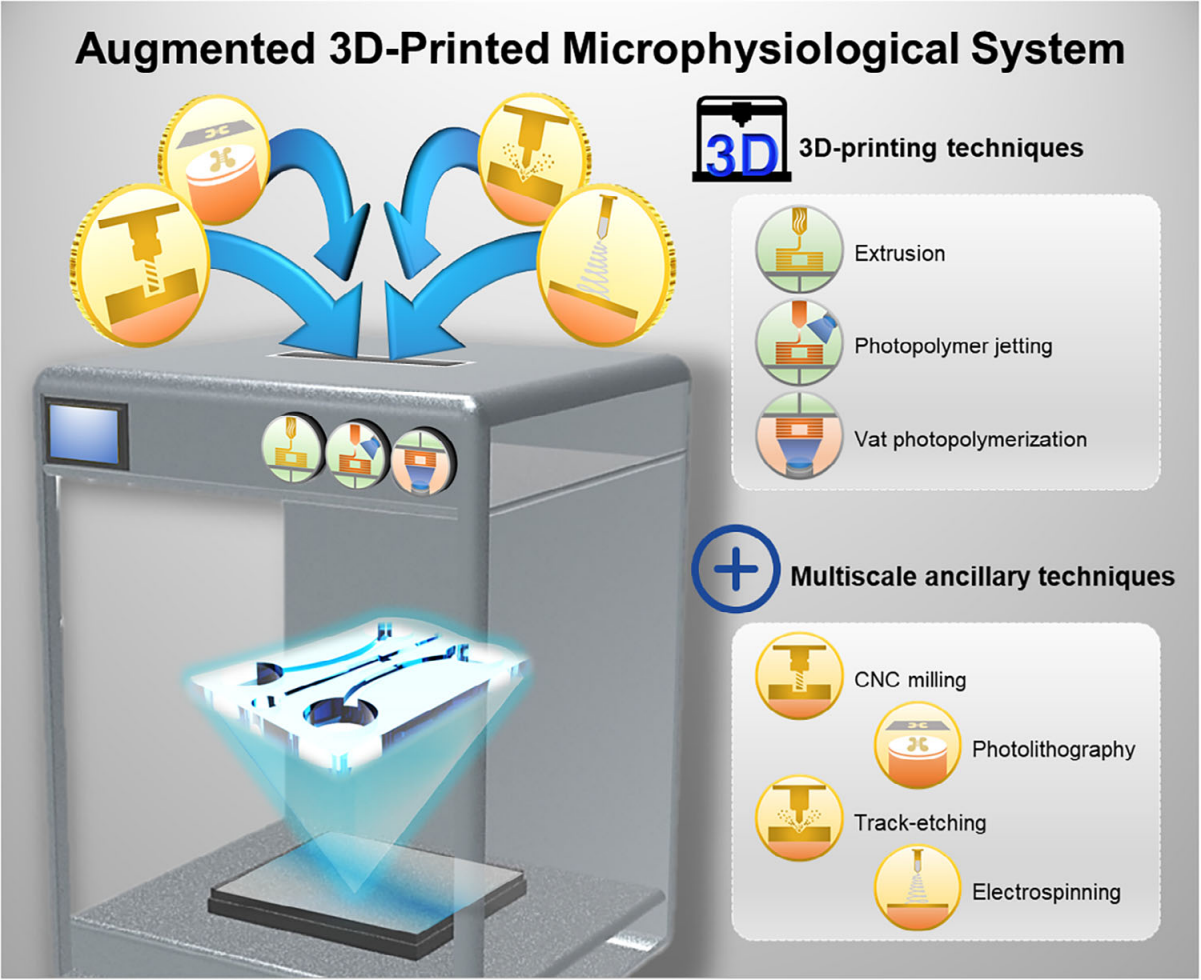
Augmented 3D‐printed microphysiological system (MPS) produced by 3D printing and multiscale ancillary techniques. Extrusion, photopolymer jetting, and vat photopolymerization are the most representative techniques for MPS production. However, MPS manufactured exclusively using 3D‐printing technology has several limitations, including sophistication and production scale. Recently, 3D‐printed MPS combined with multiscale ancillary techniques such as CNC milling, photolithography, track etching, and electrospinning have been developed to overcome these limitations.

However, there are some disadvantages to producing MPS molds or MPS chips using 3D printers compared with existing methods such as photolithography and PDMS replica molding. 3D printing has a lower resolution than photolithography, which results in poorer surface quality.^[^ ^20^ ^]^ 3D printing alone may struggle to fully replace traditional photolithography methods because MPS chip fabrication requires high resolution to manage the microenvironment at the cellular scale. The reliability of 3D‐printed MPS chips is lower than that of chips manufactured using conventional methods.^[^ ^21^ ^]^ Materials used in photopolymerization‐based 3D printing for MPS chip fabrication are often not biocompatible.^[^ ^22^ ^]^ To address these challenges, a variety of macro‐, micro‐, and nano‐fabrication techniques have been recently integrated with 3D printing as multiscale ancillary methods for overcoming limitations associated with structural complexity and scalability. This combination, which is referred to as the augmented 3D‐printing technique, offers a hybrid approach for MPS fabrication (**Figure** **1**).

**Figure 2:**
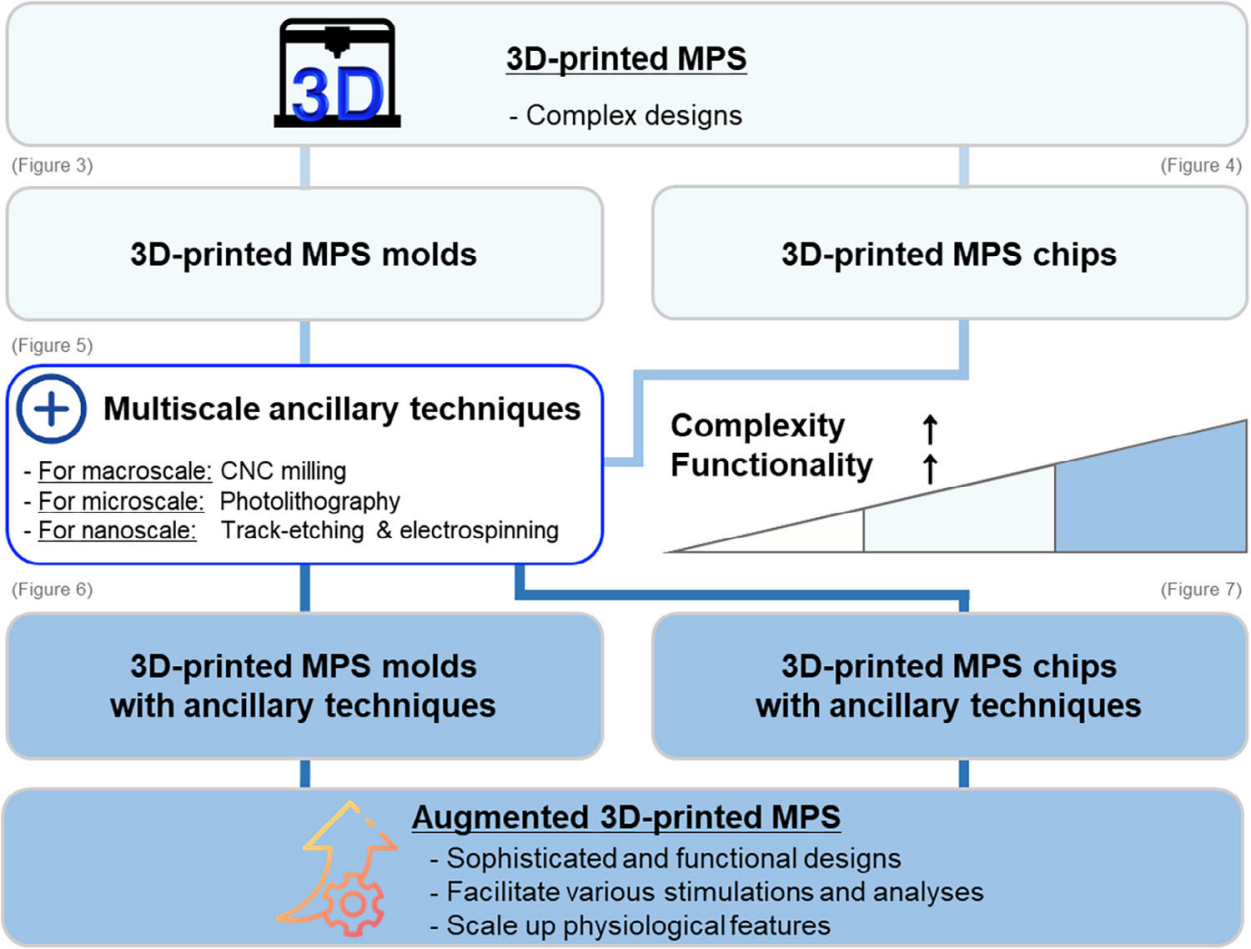
Evolutionary tree of augmented 3D‐printed MPS. 3D‐printed microscale structures are used to construct microfluidics as molds or chips in synergy with the various post‐processing steps. The 3D‐printed MPS are created by introducing different cells and cell culture microenvironments to this 3D‐printed microfluidics. In addition, augmented 3D‐printed MPS with various functionalities are formed by combining various existing multiscale ancillary techniques.

In this review, we summarize recent advances in augmented 3D‐printing techniques for MPS fabrication. We provide an overview of the 3D‐printing methods used to produce MPS and highlight the unique characteristics of each technique. Further, we reviewed studies that utilized 3D printing for preparing MPS molds or for directly fabricating MPS chips. 3D‐printed molds are used as intermediate tools to shape biomaterials such as hydrogels or elastomers; these molds enable researchers to produce soft, physiologically relevant architectures that support complex tissue microenvironments. Their strength lies in enabling the precise replication of biological topographies and compatibility with various casting‐based workflows. In contrast, 3D‐printed chips are direct‐use devices that are often designed as enclosed microfluidic systems with integrated features such as channels, chambers, or reservoirs. Further, they are engineered to support real‐time experiments, including dynamic perfusion, sensing, and long‐term cell culture. These chips are often modular and reusable, which facilitates their integration into standard laboratory setups. We aim to highlight the diversity of design approaches within the MPS field and guide readers in selecting fabrication strategies best suited to their experimental goals by distinguishing between these categories. Further, we discuss multiscale ancillary techniques that can address the limitations of current 3D‐printing methods when integrated (**Figure** **2**). Finally, we outline future perspectives on augmented 3D‐printing techniques for MPS fabrication. We believe that MPS produced through augmented 3D printing holds significant promise for fundamental biological studies, including cell biology and drug screening in pharmacology. Moreover, augmented 3D‐printed MPS have the potential to open new avenues in regenerative medicine and tissue engineering.

## 2 Conventional 3D‐Printing Techniques for the Fabrication of MPS Molds and Chips

General 3D‐printing techniques, which can be used to manufacture MPS molds or chips before delving into augmented 3D‐printed MPS using multiscale ancillary techniques, are presented. 3D printing has had a significant effect on the fields of microfluidics and MPS.^[^ ^23^ ^]^ Therefore, we describe the characteristics of each 3D‐printing technique and summarize postprinting processes before printed products can be used as MPS molds or chips (**Table** **1**).

**Table 1:** Postprocessing of 3D‐printed MPS molds/chips.

Objective	3D‐printing technique	Method	Refs.
Surface smoothing	Extrusion (FDM)	Dissolving the surface of printed parts in acetone to fill spaces between layers	[25, 29]
	Photopolymerization	Coating the surface of printed parts with a lubricant	[32]
	Photopolymerization	Sanding the surface of the printed part	[34, 44]
	Photopolymerization (DLP)	Introducing grayscale values (anti‐aliasing) to reduce the effect of staircases between layers	[35]
Prevent PDMS curing inhibition	Photopolymerization	Using PEGDA‐based photopolymers	[36, 38]
	Photopolymerization	Methylating existing photopolymers	[37]
	Photopolymerization	Irradiating UV and heat	[39]
	Photopolymerization	Coating the surface of printed parts with parylene	[40a]
	Photopolymerization	Coating the surface of printed parts with PMMA	[40b]
	Photopolymerization	Coating the surface of printed parts with SU‐8	[40c]
Reduce cell toxicity	Photopolymerization	Irradiating UV and heat	[30, 136]
	Photopolymerization	Coating the surface of printed parts with parylene	[40, 137]
Bonding with glass	Extrusion	Thermal bonding on a glass surface	[41]
	Extrusion	Using melted parafilm as an adhesive	[42]
	Photopolymerization	Using a liquid‐state PDMS as an adhesive	[44]
	Photopolymerization	Using photocurable glue	[45]
	Photopolymerization	Coating printed parts with PDMS and treating them with plasma for a covalent bond	[47]
Bonding with adhesive tape	Photopolymerization	Load printed parts onto the adhesive tape and apply pressure	[19a,b]

### 2.1 Fabrication of MPS Molds and Chips via Extrusion‐Based 3D Printing

Extrusion‐based 3D printing, namely, fused deposition modeling (FDM), is widely used for fabricating MPS.^[^ ^24^ ^]^ In FDM, thermoplastic polymer filaments such as acrylonitrile, 1,3‐butadiene, styrene (ABS),^[^ ^25^ ^]^ polylactic acid,^[^ ^26^ ^]^ poly(methyl methacrylate) (PMMA),^[^ ^27^ ^]^ and polystyrene^[^ ^28^ ^]^ are melted in a heated nozzle and deposited layer‐by‐layer to build 3D structures. However, the printed surfaces are rough because of the layer‐by‐layer deposition of discrete lines, which leads to light scattering and poor optical transparency. Consequently, FDM suffers from a lower spatial resolution than light‐based printing methods. To address this limitation, post‐processing with solvents such as acetone can be applied to smooth the surface and enhance optical clarity. Acetone treatment can be used to selectively melt specific regions to form microchannels.^[^ ^25^, ^29^ ^]^ Unlike photopolymer‐based printing, FDM‐printed materials are biocompatible without further treatment, making them suitable for biological applications.^[^ ^30^ ^]^

### 2.2 Fabrication of MPS Molds and Chips via Photopolymerization‐Based 3D Printing

Photopolymerization uses a photocurable resin solidified layer‐by‐layer through selective exposure to ultraviolet (UV) light. This technique is classified into photopolymer jetting, wherein the resin is selectively deposited onto specific regions, and vat photopolymerization, wherein the surface of the resin bath is selectively irradiated with UV light. Compared with extrusion‐based methods, photopolymerization offers a significantly higher printing resolution and smoother surface finish, thereby making it well‐suited for fabricating fine microstructures. However, unlike extrusion, which supports a wide variety of thermoplastic filaments for diverse applications, photopolymerization is limited to photosensitive polymers. In addition, the use of UV light and photoinitiators raises concerns regarding potential material degradation and limited biocompatibility, particularly in applications requiring prolonged contact with biological components. These trade‐offs between fabrication precision and material versatility need to be carefully considered when selecting an appropriate MPS construction method.

#### 2.2.1 Photopolymer Jetting

Photopolymer jetting involves spraying a liquid photocurable material onto a specific area and curing it with a UV lamp. Unlike vat photopolymerization, which prints one type of photopolymer at a time, photopolymer jetting enables the simultaneous printing of several types of photopolymers using multiple nozzles, thereby making it possible to fabricate multimaterial microfluidic devices.^[^ ^31^ ^]^ However, photopolymer jetting results in greater surface roughness than vat photopolymerization because of the spraying method. This surface roughness can contribute to the nonuniformity of the flow velocity and shear rate within the microchannel. The surface roughness of microchannels produced by photopolymer jetting can be improved by applying a lubricating coating.^[^ ^32^ ^]^ In addition to enhancing the surface smoothness, this coating helps achieve a uniform flow velocity and shear rate.

#### 2.2.2 Vat Photopolymerization

Vat photopolymerization selectively projects UV light into the vat of a liquid photocurable resin for building a layer on a support plate. The object formed layer‐by‐layer as the plate moved upward. The process is referred to as stereolithography when a UV laser cures the resin in dot units; when a digital micromirror device cures an entire plane at once, it is referred to as digital light processing (DLP). The 3D printer resolution and surface smoothness are considered superior in the following order: vat photopolymerization, photopolymer jetting, and extrusion.^[^ ^33^ ^]^ However, the resolution of these 3D‐printing methods is still low compared to that of other fabrication processes. A sanding process is often applied to enhance resolution.^[^ ^34^ ^]^ Recent advancements in DLP technology have introduced grayscale values instead of just black and white (anti‐aliasing) values, which reduces the staircase effect between the layers of the 3D‐printed part.^[^ ^35^ ^]^

After photopolymerization, post‐processing is required before printed parts can be used as MPS or MPS chips. Residual monomers and photoinitiators in printed parts can inhibit PDMS curing, which prevents its immediate application in MPS molds or chips.^[^ ^36^ ^]^ In addition, photopolymers are cytotoxic because of the presence of photoinitiators, making them unsuitable for direct use as MPS chips.^[^ ^37^ ^]^ To address these limitations, we explored two strategies. The first strategy involves developing new photopolymers that can completely cure the PDMS. For example, photopolymers that can fully cure PDMS have been fabricated using polyethylene glycol diacrylate (PEGDA) as the primary raw material^[^ ^36^, ^38^ ^]^ and methylating existing photopolymers.^[^ ^37^ ^]^ The second strategy focuses on postprocessing to remove inhibitory residues from printed parts. Techniques such as UV irradiation or heat treatment can eliminate uncured monomers and photoinitiators; however, heat may deform the structure.^[^ ^39^ ^]^ Alternatively, surface coating with materials such as parylene, PMMA, or SU‐8 can prevent direct contact between the PDMS and inhibitory substances.^[^ ^40^ ^]^

### 2.3 Various Bonding Methods Between 3D‐Printed Parts and Flat Substrates

Although some 3D‐printed parts are fabricated as monolithic structures that can contain closed microchannels, most MPSs are manufactured by bonding a flat substrate to a 3D‐printed surface. Therefore, the bonding method between the 3D‐printed component and flat substrate is a critical factor in the 3D printing of MPS chips. Thermal bonding is often employed for bonding 3D‐printed parts produced by extrusion into glass, wherein the surface of the printed part acts as a thermoplastic adhesive when the temperature at the interface between the 3D‐printed part and glass is raised.^[^ ^41^ ^]^ An alternative approach is to place a melted parafilm between the printed part and the glass, using parafilm as an adhesive to bond the two surfaces.^[^ ^42^ ^]^

Alternative bonding methods are required for photopolymerization instead of thermal treatment because photopolymerized parts have a cross‐linked molecular structure, unlike thermoplastics.^[^ ^43^ ^]^ One approach involves placing a 3D‐printed part on a liquid‐state spin‐coated PDMS substrate and curing the PDMS to produce an MPS device with a thin PDMS substrate.^[^ ^44^ ^]^ Similarly, PDMS can be applied to the surface of a 3D‐printed part and used as an adhesive to bond the part to glass.^[^ ^44b^ ^]^ Another bonding method uses photocurable glue as an alternative to PDMS for integrating 3D‐printed parts with glass.^[^ ^45^ ^]^ In addition, biocompatible adhesive tape can serve as a flat substrate for 3D‐printed MPS chips.^[^ ^19a,b^ ^]^ In addition to adhesive‐based techniques, plasma treatment is used as a bonding method for utilizing the covalent bonding principle between plasma‐treated PDMS and glass.^[^ ^46^ ^]^ Further, it is possible to bond the printed part with glass by spraying silicon onto the surface of the 3D‐printed part followed by plasma treatment.^[^ ^47^ ^]^

## 3 3D‐Printed MPS Mold/Chip Without Multiscale Ancillary Techniques

### 3.1 MPS Molds Without Multiscale Ancillary Techniques

MPS molds fabricated using 3D‐printing techniques tend to exhibit greater geometrical complexity than those produced by conventional photolithography. This technique enables the fabrication of structures with variable heights in a single process, as well as structures with curved shapes. In contrast, such features are either difficult to achieve or entirely infeasible using traditional photolithographic methods, which are limited to planar‐layered designs.

#### 3.1.1 3D‐Printed MPS Molds for Microchannels

**Figure 3:**
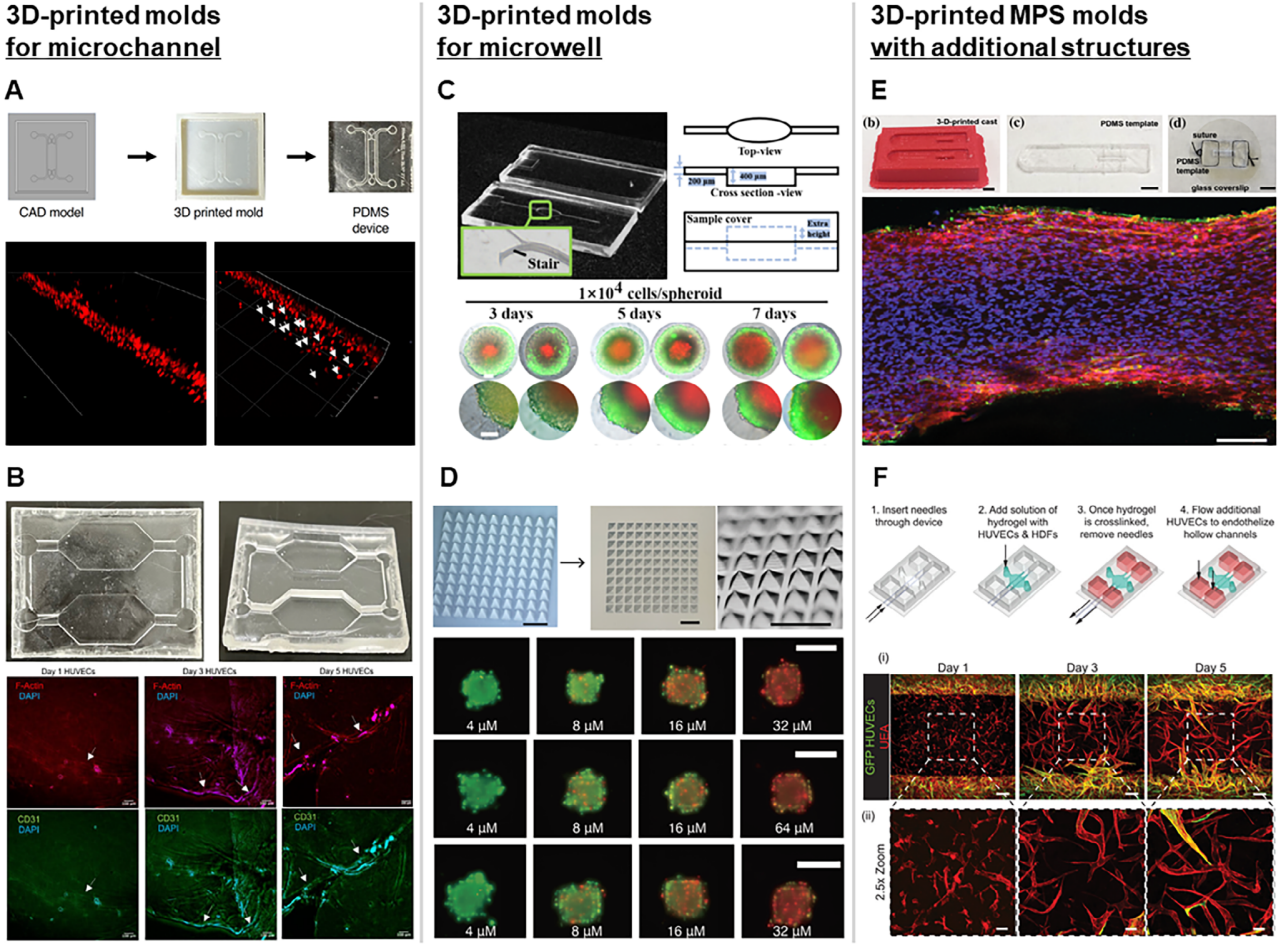
Representative examples of MPS fabricated using 3D printed molds. A) MPS incorporates three microchannel layers of distinct heights to facilitate mold demolding, selective drug loading, and cell culture. This fabricated structure enables reliable PDMS replication and supports 3D cancer cell invasion and drug response assays. Reproduced from Poskus et al. under the terms of the CC‐BY license.^[^ ^48^ ^]^ B) A capillary circuit chip models in vitro cardiovascular tissue by integrating stenotic and healthy blood vessel geometries. Thrombus formation occurs preferentially at the stenotic site under whole blood perfusion, which simulates pathological clotting conditions. Reproduced from Esparza et al. under the terms of the CC‐BY license.^[^ ^49^ ^]^ C) A microwell array comprises oblong, tapered wells optimized for trapping uniform brain cell spheroids. This platform integrates sequential isolation, staining, and embedding steps, thereby enabling the efficient preparation of brain assemblies for downstream analysis. Reproduced from Ning et al. with permission.^[^ ^51^ ^]^ Copyright 2024, ELSEVIER. D) A modular MPS platform comprising a spheroid capture chamber aligned with a micropipette inlet, which enables the localized injection of reagents into selected spheroids. This configuration enables the precise manipulation of microenvironments within individual organoid units. Reproduced from Qin et al. with permission.^[^ ^52^ ^]^ Copyright 2024, AMERICAN CHEMICAL SOCIETY. E) This MPS includes the silk suture. In this MPS, a muscle bundle is anchored to a structure of silk to maintain its shape. Reproduced from Ariyasinghe et al. with permission.^[^ ^53^ ^]^ Copyright 2021, Kluwer Academic Publishers. F) This is designed to make lumens in MPS by removing microneedles after loading the hydrogel. The vascularized hepatic MPS is reproduced using this luminal structure. Reproduced from Song et al. with permission.^[^ ^54^ ^]^ Copyright 2021, WILEY.

Making molds for MPS chips with microchannels of different heights using photolithography is time‐consuming and labor‐intensive. However, such complex structures can be easily produced using 3D‐printing techniques. Poskus et al. developed molds with different heights to utilize microchannels for gradient generators and microrails via vat photopolymerization.^[^ ^48^ ^]^ This MPS device was used to confirm the distribution of invading cells in the 3D collagen hydrogel, which enables the real‐time monitoring of single‐cell invasion. However, the resolution of the system was insufficient for fabricating features on the scale of tens of microns, which can limit its applicability to certain single‐cell analyses (**Figure** **3**A).

3D curved structures cannot be produced by photolithography; however, they can be easily created by 3D printing. PDMS replica molding with a 3D‐printed MPS mold featuring a curved surface enabled the production of MPS chips with complex structures, such as vascular tissues. Esparza et al. developed a mold for 3D curved microchannels using vat photopolymerization to replicate vascular tissue models in PDMS channels.^[^ ^49^ ^]^ In their study, human umbilical vein endothelial cells (HUVECs) cultured in MPS chips exhibited both curved and disjointed capillary‐like structures as well as elongated, continuous tubes with a circular orientation. Further, capillary‐like lumen formation was observed in the device (Figure 3B). However, the device did not incorporate co‐culture conditions, limiting its ability to replicate physiologically relevant multicellular interactions such as endothelial–cardiomyocyte crosstalk.

#### 3.1.2 3D‐Printed MPS Molds for Microwell

Various 3D‐printing techniques such as vat photopolymerization and photopolymer jetting have enabled fabricating deep wells and channel structures for spheroid formation. Studies have also demonstrated that cell spheroids of different sizes can spontaneously form within microwells or hydrogels assisted by surface tension or growth factor gradients.^[^ ^15^, ^50^ ^]^ These structures support high‐throughput and size‐variable spheroid generation within MPS platforms.

Molds of various designs were constructed for fabricating structures that can control cell spheroid formation. Ning et al. developed a microwell array using embedded agar.^[^ ^51^ ^]^ This design prolonged the culture time and decreased cell death in the center region of the spheroids. Agar embedding can be performed on‐chip to ensure the location and orientation of 3D spheroids, which facilitates a more precise investigation of their heterogeneous cellular architecture. This approach overcomes the limitations of existing spheroid culture methods that rely on manual placement (Figure 3C). A remaining challenge is the difficulty in visualizing the fusion interface between two spheroids after sectioning, largely because of the inconsistent slicing planes caused by the spatial constraints of the embedding chamber. Using vat photopolymerization, Qin et al. produced a platform comprising pyramidal microchambers designed to capture single cells and form cell spheroids, which positions them at the center of the MPS device.^[^ ^52^ ^]^ In addition, this MPS chip successfully performed a dose‐response assay using flow cell‐containing spheroids for three antitumor drugs, Sorafenib, Oxaliplatin, and Docetaxel (Figure 3D). However, wells smaller than 150 µm in diameter exhibited noticeable shape distortion because of the resolution limits of the printer, indicating that features below 100 µm can require fabrication using traditional soft lithography techniques.

#### 3.1.3 3D‐Printed MPS Molds with Additional 1D Structures

One advantage of 3D‐printing molds is their ability to incorporate thin, elongated structures such as sutures or needles, with diameters of several hundred millimeters, after mold fabrication. Ariyasinghe et al. produced a mold by extrusion to form 3D skeletal muscle.^[^ ^53^ ^]^ The mold had a 2‐mm‐deep structure designed to load a cell‐laden hydrogel and featured cylindrical holes for microneedle insertion. After inserting silk sutures into these cylindrical holes, PDMS replica molding was performed on the mold for creating an MPS chip with the corresponding cylindrical holes. Aligned muscle bundles anchored to the silk sutures were formed by reinserting silk sutures into these cylindrical holes and applying the myoblast‐laden hydrogel (Figure 3E). However, limited nutrient diffusion leads to the peripheral localization of myotubes and physiologically suboptimal tissue formation. Similarly, Song et al. developed a mold for 3D vascular networks in MPS using vat photopolymerization.^[^ ^54^ ^]^ This mold included cylindrical holes for microneedle insertion. A cell‐laden hydrogel with two parallel lumens was formed after inserting microneedles into cylindrical holes, loading the cell‐laden hydrogel, and removing microneedles after crosslinking. Endothelial cells, fibroblasts, and hepatocyte‐laden hydrogels were loaded onto the MPS chip to produce a vascularized hepatic MPS (Figure 3F).

### 3.2 MPS Chip Without Multiscale Ancillary Techniques

When a mold is used to fabricate an MPS chip, only identical and relatively simple features can be reproduced, which limits the design possibilities of the MPS chip. However, producing an MPS chip with a more complex geometry is possible if a 3D‐printed component is directly used as an MPS chip. The following section presents MPS devices with various internal structures, including simple microchannels, chambers with various internal structures, and complex geometric structures, such as 3D meshes.

#### 3.2.1 3D‐Printed Microchannels

**Figure 4:**
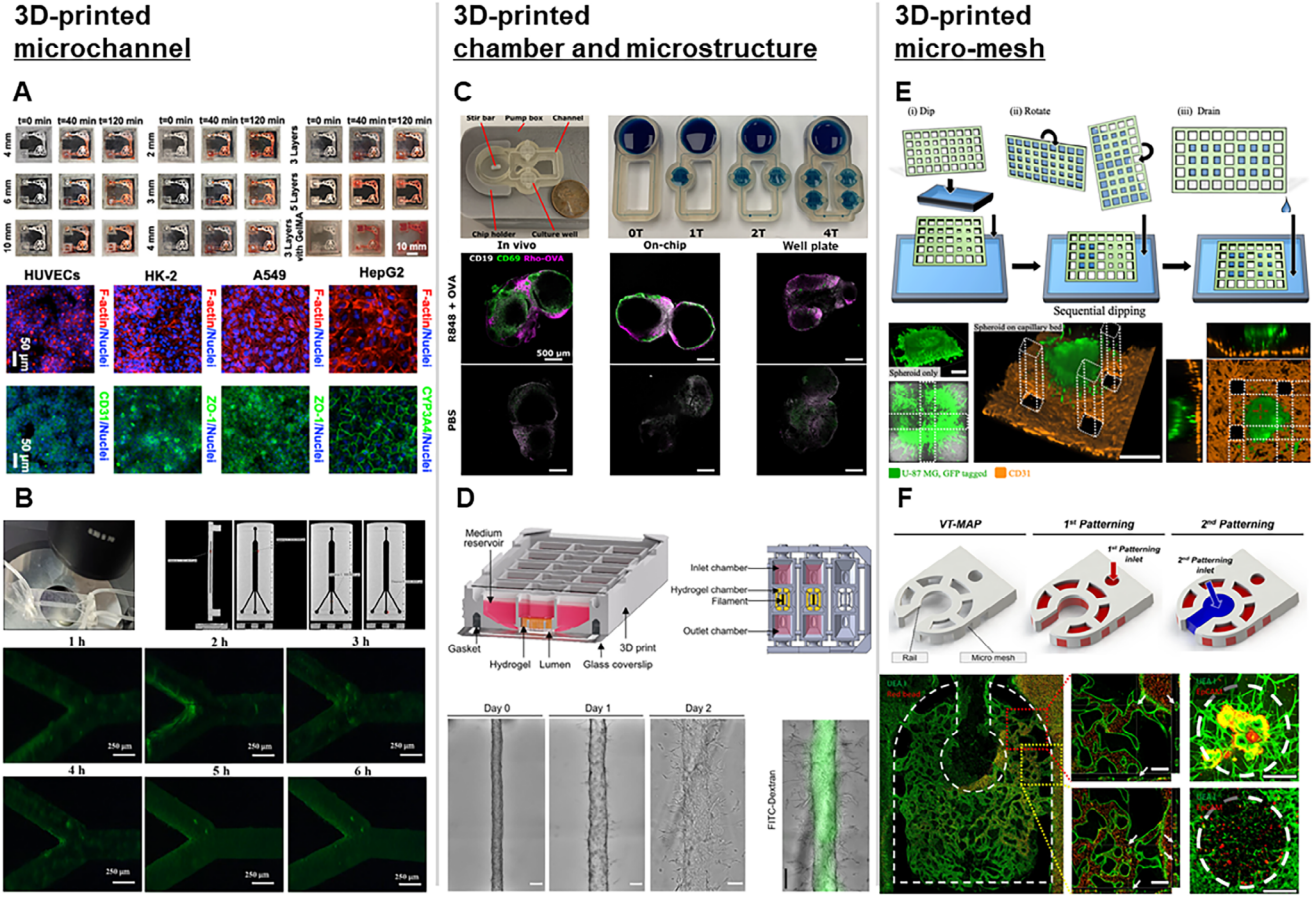
Representative examples of 3D‐printed MPS chips. A) The MPS chip is manufactured for autonomous, capillary‐driven media delivery to multiple tissue compartments. Organ‐specific cells, including hepatocytes and tumor cells, are cultured on integrated paper scaffolds, which enables metabolism‐dependent drug response assessment without external power. Reproduced from Li et al. with permission.^[^ ^55^ ^]^ Copyright 2022, Royal Society of Chemistry. B) An MPS chip supports the real‐time monitoring of bacterial growth under different flow conditions. GFP‐expressing *E. coli* cultured in parallel channels exhibited distinct adhesion and biofilm formation patterns when exposed to varying media compositions and shear flow. Reproduced from Ashish et al. with permission.^[^ ^56^ ^]^ Copyright 2024, ELSEVIER. C) This MPS is manufactured with integrated pumping structures, enabling directional fluid flow without external tubing. Communication between upstream and downstream compartments is modeled for lymph node activation and CD69 induction under vaccine stimulation. Reproduced from Cook et al. under the terms of the CC‐BY‐NC‐3.0 license.^[^ ^60^ ^]^ D) The MPS supports vessel formation from hPSC‐derived endothelial cells. Shear stress promoted angiogenic sprouting and endothelial barrier formation within the dual open‐chamber design. Reproduced from Remmert et al. under the terms of the CC‐BY‐4.0 license.^[^ ^61^ ^]^ E) The MPS is composed of meshes with varying window sizes. The cell‐laden hydrogel can be patterned selectively based on the mesh window size. This enables co‐cultures in which different types of cells are spatially separated from one another. Reproduced from Lee et al. under the terms of the CC‐BY‐4.0 license.^[^ ^63^ ^]^ F) A mesh‐supported MPS chip featuring open lattice wells facilitates a vascularized organoid culture with a controllable size and ECM composition. Reproduced from Lee et al. under the terms of the CC‐BY‐NC‐3.0 license.^[^ ^65^ ^]^

Although microchannels manufactured through PDMS replica molding in 3D‐printed MPS molds can have complex shapes, they require bonding to a flat substrate. In contrast, methods that directly utilize MPS‐embedded microchannels, such as 3D printing onto a flat substrate or the fabrication of monolithic structures via 3D printing, eliminate the need for substrate bonding. Li et al. created a microchannel by the extrusion‐mediated printing of materials directly onto paper.^[^ ^55^ ^]^ The siphon characteristics of multi‐organ‐on‐paper platforms were optimized by varying structural configurations, including printed layer numbers, medium reservoir heights, evaporation opening diameters, and presence of the GelMA hydrogel, at different time points after initiating the medium flow. After seeding cells through the microchannel, the biological functions of corresponding organ mimics were evaluated by examining the behavior of HepG2, A549, HK‐2 cells, and HUVECs (**Figure** **4**A). Despite their cost‐effectiveness and simplicity, paper‐based platforms inherently suffer from the nonspecific adsorption of biomolecules and drugs, which can interfere with fluorescence imaging and skew drug dosing measurements. Ashish et al. developed MPS chips on supporting structures that included microchannels.^[^ ^56^ ^]^ On the chip, the growth kinetics of bacteria increased at higher flow rates when both types of media were used. In addition, bacterial growth and adhesion were enhanced, and uniform biofilm formation was observed in channels after incubation with the two media and GFP‐expressing *Escherichia coli* (BL21) cells (Figure 4B).

#### 3.2.2 3D‐Printed Chambers and Microstructures Inside the Chamber

3D‐printing technology can produce simple wells or channel structures as well as more complex geometries. This approach enables the simultaneous production of a chamber tailored to the purpose of the study as well as multiple microstructures within the chamber. However, most MPS devices manufactured in this manner are not monolithic, requiring an additional bonding process between the 3D‐printed device and a flat surface.

When reconstructing in vitro neural circuits, axonal isolation is necessary for spatially controlled stimulation and observation.^[^ ^7^, ^57^ ^]^ Several studies utilized 3D‐printed MPS for axon isolation. Wardyn et al. developed an MPS chip that can achieve axon isolation using extrusion and bonded the 3D‐printed chip to a coverslip using biocompatible glue.^[^ ^42^ ^]^ This device features a structure that separates the axon from the chamber where neurons are seeded. The chamber was bonded to a coverslip, and the axon separation structure was positioned several micrometers above the coverslip. This narrow gap prevents the soma of the neuron from entering, while enabling the axon to pass through. Johnson et al. fabricated an MPS chip for axon isolation via extrusion.^[^ ^58^ ^]^ This chip was 3D‐printed directly onto a coverslip in three steps: printing the microchannels for axonal guidance, a sealant layer for preventing fluid exchange, and a chamber for cellular patterning. The design of this device is similar to that of the Campenot chamber.^[^ ^59^ ^]^ Different cell types can be loaded, and various growth factors can be applied to each chamber using microchannels, thereby enabling axons to grow into adjacent chambers.

The immune function of the lymph nodes is reproduced through communication between the lymph nodes and upstream drainage sites. Cook et al. fabricated a multicompartment MPS chip using extrusion‐based 3D printing.^[^ ^60^ ^]^ The chip included a microchannel and microchamber designed for co‐culturing multiple organ tissues to evaluate the immune function of the lymphatic tissue. This platform enables comparison of acute immune responses among in vivo models, on‐chip systems, and well plates. Notably, both on‐chip and in vivo vaccinations induced strong CD69 expression, particularly within the CD19⁺ region, whereas minimal responses were observed in phosphate‐buffered saline‐treated controls (Figure 4C). However, the current system exhibits insufficient interstitial fluid flow and shear stress within tissue slices because of the existing mesh geometry and flow parameters. These values were substantially lower than the physiological ranges observed in lymph nodes, potentially compromising the biological relevance of modeled immune responses.

If an MPS device is manufactured with the same specifications as a standardized cell culture plate, it can be made compatible with a wide range of existing cell culture systems, which include multipipettes and microscopes. Kim et al. introduced a 96‐well plate MPS device with a fluid‐guide structure inside each well, and this device was fabricated using vat photopolymerization.^[^ ^19b^ ^]^ After 3D printing, the device did not have a bottom surface. Therefore, it was attached to a transparent and biocompatible adhesive tape that served as an alternative substrate. This design enabled precise hydrogel patterning with the assistance of a fluid‐guiding structure within each well. This study demonstrated that angiogenesis varies among different cancer types. Remmert et al. fabricated a microchannel with two open chambers using vat photopolymerization and bonded it to a coverslip using an adhesive foil.^[^ ^61^ ^]^ Human pluripotent stem cell‐derived endothelial cells were loaded into an open microchamber, and endothelial cells were cultured within microchannels. The study demonstrated that vascular lumens were formed, and shear flow induced sprouting angiogenesis, thereby leading to the development of an endothelial barrier (Figure 4D). This system supports vascular morphogenesis but relies on gravity‐driven flow, generating subphysiological shear stress (≈3 dyne/cm^2^). In addition, collagen hydrogel degradation by endothelial cells limits long‐term culture and requires more stable hydrogel formulations.

#### 3.2.3 3D‐Printed Micro‐Mesh

Although 3D‐printing techniques enabled the creation of complex structures, their applications have been largely limited to fabricating simple microchannels for fluid perfusion and chambers for hydrogel encasement. A new 3D mesh structure is proposed as a potential solution to develop a macroscale 3D cell culture platform.^[^ ^62^ ^]^

Lee et al. fabricated a 3D orthogonal mesh structure using vat photopolymerization.^[^ ^63^ ^]^ This mesh structure featured windows of varying sizes. The solution drained out of the mesh through larger windows while remaining within smaller windows when the mesh structure was dipped into a liquid hydrogel solution and removed. Size‐dependent patterning is based on the pressure difference required for liquid leakage.^[^ ^64^ ^]^ This design enables various types of cells to be patterned for small and large windows of the mesh structure (Figure 4E). Discrepancies arose between the designed and printed structures because of the limited resolution of the DLP 3D printer, particularly at smaller gap sizes, which results in deviations from theoretical predictions.

Lee et al. produced a micromesh structure with the potential to be used as a 3D curved MPS via vat photopolymerization.^[^ ^65^ ^]^ The micromesh structure was fabricated as an open lattice for restricting the fluid spreading and enabling precise patterning within desired regions, effectively preventing flow into the central area. The cell‐laden fibrinogen hydrogel became entrapped within the windows of the curved mesh, enabling cultivation of various cell types in both the central and side channels (Figure 4F).

A summary of the representative examples of 3D‐printing methods and cell sources introduced in a 3D‐printed MPS mold/chip without multiscale ancillary techniques is provided in **Table** **2**.

**Table 2:** 3D‐printed MPS molds/chips without multiscale ancillary techniques.

Refs.	3D‐printing method	Tissue or organ type	Cell types	Cell culture environment
	‐Device features			
	‐Results			
3D‐printed MPS molds without multiscale ancillary techniques – Microchannel				
Poskus et al.^[^ ^48^ ^]^	Vat photopolymerization	Tumor tissue	MDA‐MB‐231 (breast cancer cells) HUVECs	3D (In hydrogel)
	High‐resolution 3D‐printed molds for PDMS microchannels filled with collagen ECM			
	Reduced cancer cells invasion by treating doxorubicin in a dose‐dependent manner			
Esparza et al. ^[^ ^49^ ^]^	Vat photopolymerization	Cardiovascular tissue	HUVECs Cardiac fibroblasts	3D (On 3D PDMS inner surface)
	Fully 3D‐printed capillary circuit microfluidic chip			
	Perfusion culture supported vascular‐like structure formation by co‐cultured cells			
3D‐printed MPS molds without multiscale ancillary techniques – Microwell				
Fang et al. ^[^ ^50a^ ^]^	Vat photopolymerization	Breast cancer	MCF‐7 (breast cancer cells) Dermal fibroblasts	3D (Cell spheroid)
	Producing tumor spheroids of different sizes on a single chip			
	Higher chemo‐resistance in large tumor spheroids than in small ones.			
Kamei et al. ^[^ ^15a^ ^]^	Photopolymer jetting	Stem cell niche	hESCs	3D (Cell spheroid in hydrogel)
	Fabrication of a hard high channel with photolithography			
	The higher the concentration of the growth factor, the larger the formed stem cell spheroids			
Ruppen et al. ^[^ ^50b^ ^]^	Vat photopolymerization	Lung cancer	Primary lung epithelial tumor cells Primary pericytes	3D (Cell spheroid)
	From tumor spheroid formation to drug treatment and observation on a single chip			
	Co‐cultured tumor spheroids are more chemo‐resistant than monocultures			
Ning et al. ^[^ ^51^ ^]^	Vat photopolymerization	Tumor tissue	HepG2 HeLa	3D (Cell spheroid in hydrogel)
	3D‐printed mold for PDMS chip enabling spheroid isolation			
	Integrated on‐chip trapping, fluorescent staining, and matrix embedding of cell spheroids			
Qin et al. ^[^ ^52^ ^]^	Vat photopolymerization	Tumor tissue	Hep3B	3D (Cell spheroid)
	3D‐printed microwell arrays for spheroid formation and drug screening			
	Dynamic combinatorial drug assays with sequential flow, which reveals drug order‐dependent efficacy			
3D‐printed MPS molds without multiscale ancillary techniques – With additional 1D structures				
Ariyasinghe et al. ^[^ ^53^ ^]^	Extrusion	Muscle tissue	C2C12 (Myoblasts)	3D (Muscle bundle)
	Inserting a suture to anchor a muscle bundle			
	Formation of contractile muscle bundle that responds to electrical stimulation			
Song et al. ^[^ ^54^ ^]^	Vat photopolymerization	Vascularized hepatic tissue	Primary human hepatocytes HUVECs Dermal fibroblasts	3D (In hydrogel)
	Forming a lumen structure in a hydrogel using a microneedle for the adhesion of endothelial cells			
	Formation of vascularized hepatocytic tissue that can synthesize hepatic proteins such as albumin			
3D‐printed MPS chips without multiscale ancillary techniques – Microchannel				
Li et al. ^[^ ^55^ ^]^	Extrusion	Multiorgan (vasculature, liver, tumor, and kidney)	HUVECs HepG2 A549 HK‐2	2D (On a paper substrate)
	Multiorgan‐on‐paper platform utilizing capillary and evaporation‐driven forces for passive media flow			
	Cytotoxicity and metabolism‐dependent efficacy of drugs across different organ models			
Ashish et al. ^[^ ^56^ ^]^	Extrusion	Bacteria	*Escherichia coli*	2D (On glass)
	3D‐printed microfluidic device with controlled microenvironments			
	Bacterial growth dynamics under different flow conditions			
3D‐printed MPS chips without multiscale ancillary techniques – Chamber and microstructure				
Wardyn et al. ^[^ ^42^ ^]^	Extrusion	Neural circuit	Primary hippocampal neurons	2D (On glass)
	Only neurites pass through the low‐height channel.			
	Observations of only neurites without cell bodies			
Johnson et al. ^[^ ^58^ ^]^	Extrusion	Neural circuit	Primary hippocampal neurons Primary ganglia cells Primary Schwann cells	2D (On glass)
	Hierarchical structure with a microchannel for axonal guidance and a chamber for cell patterning			
	Observation of the axonal transport of the virus in an aligned neural circuit			
Cook et al. ^[^ ^60^ ^]^	Vat photopolymerization	Lymph node	Murine lymph node slices	3D (On mesh)
	Multicompartment organ‐on‐chip with integrated tubing‐free impeller pump			
	Antigen drainage and immune activation comparable to in vivo responses			
Ko et al. ^[^ ^19a^ ^]^	Vat photopolymerization	Brain cancer	U87MG (Glioblastoma cells) HUVECs Lung fibroblasts	3D (Cell spheroid in hydrogel)
	Fabrication of open‐microchannel using fluid‐guide rail structure in a 96‐well plate format			
	Verifying the efficacy of antiangiogenic cancer drugs			
Kim et al. ^[^ ^19b^ ^]^	Vat photopolymerization	Colorectal cancer	SW480 (Colorectal cancer cells) HUVECs Lung fibroblasts	3D (In hydrogel)
	Fabrication of open‐microchannel using a fluid‐guide rail structure in a 96‐well plate format			
	Verifying the efficacy of antiangiogenic cancer drugs			
Remmert et al.^[^ ^61^ ^]^	Vat photopolymerization	Vessel	iPSC‐derived endothelial cells	3D (In hydrogel)
	Fabrication of connected open chambers and microchannels			
	Formation of vascular lumen under flow conditions			
3D‐printed MPS chips without multiscale ancillary techniques – Micro‐mesh				
Lee et al.^[^ ^65^ ^]^	Vat photopolymerization	Vascularized tumor tissue	HUVECs Lung fibroblasts Tumor organoids	3D (In hydrogel)
	Fabrication of mesh‐supported open‐lattice microfluidic chip			
	Verifying organoid size‐specific responses to anti‐tumor drugs			
Lee et al.^[^ ^63^ ^]^	Vat photopolymerization	Brain cancer	U87MG (Glioblastoma cells) HUVECs Lung fibroblasts	3D (In hydrogel)
	Cell patterning by putting mesh structures with windows of varying sizes in a liquid state of cell‐laden hydrogels and then pulling them out.			
	Formation of vascularized brain cancer			
Dong et al.^[^ ^138^ ^]^	Vat photopolymerization	Liver tissue	HepG2 (Liver cancer cells)	2D (On the surface of the 3D‐printed part)
	Fabrication of a nanoporous hierarchically mesh structure using polymerization‐induced phase separation			
	3D mesh with nanopores adheres more cells than a 3D mesh without nanopores			

## 4 Multiscale Ancillary Techniques

**Figure 5:**
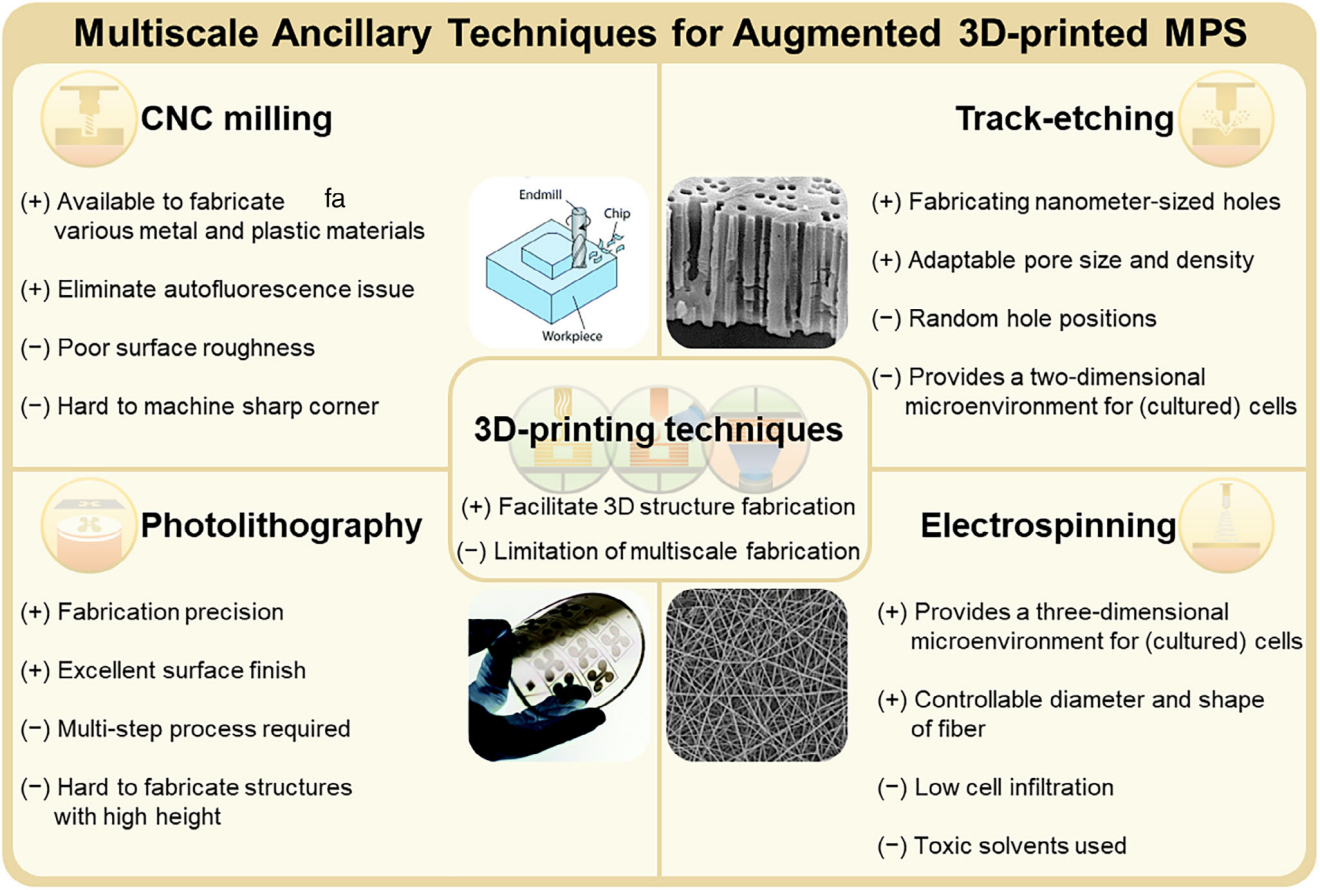
Various methods for multiscale ancillary techniques. For MPS fabrication, 3D printing techniques are excellent as they can aid in producing 3D structures with a scale ranging from several hundred micrometers to several centimeters. However, the size of the structures is limited. The multiscale MPS introduces ancillary techniques to overcome this problem. CNC milling, photolithography, track‐etching, and electrospinning are representative examples of multiscale ancillary techniques. (CNC milling–Reproduced from Guckenberger et al. with permission.^[^ ^67^ ^]^ Copyright 2015, Royal Society of Chemistry; Photolithography–Reproduced from Kang et al. with permission.^[^ ^70b^ ^]^ Copyright 2017, The Royal Society of Chemistry; Track‐etching–Reproduced from Apel et al. with permission.^[^ ^75b^ ^]^ Copyright 2001, ELSEVIER; and Electrospinning–Reproduced from He et al. under the terms of the CC‐BY‐NC‐3.0 license).^[^ ^78^ ^]^

In this section, we introduce multiscale ancillary techniques that enable augmented 3D‐printed MPS. Various macro‐, micro‐, and nano‐fabrication techniques are discussed in the following subsections (**Figure** **5**).

### 4.1 Multiscale Ancillary Techniques for Macro‐Scale Fabrication

The exclusive use of 3D printing is often impractical because of the lengthy processing time required for fiber‐by‐fiber or layer‐by‐layer stacking to fabricate large‐scale MPS molds or chips. The MPS structures can become large and complex when they incorporate various elements.^[^ ^66^ ^]^ The combination of 3D printing with macrofabrication techniques, such as milling, can offer an effective solution. Milling is a subtractive process that uses cutting tools, such as end mills, to remove materials from bulk substrates.^[^ ^67^ ^]^ Sophisticated machining is possible when performing computer numerical control (CNC) milling. However, milling often leaves tool marks on the surface, which affects surface roughness, and it is challenging to machine sharp corners in microchannels because of the curvature of the end mill tip.^[^ ^68^ ^]^ Therefore, integrating 3D printing with milling can be complementary, which enables efficient large‐scale MPS fabrication. For example, Behroodi et al. manufactured a channel structure with a width of several hundred micrometers and a length on the centimeter scale by combining 3D‐printing and CNC milling techniques.^[^ ^40b^ ^]^

### 4.2 Multiscale Ancillary Techniques for Micro‐ and Nano‐Scale Fabrication

Although the XY resolution of vat photopolymerization 3D printing is higher than that of extrusion‐based 3D printing, it is limited to a few tens of micrometers. The integration of micro‐ and nanofabrication techniques such as photolithography, PDMS replica molding, etching, and electrospinning could help address these limitations. These techniques can be effectively used to fabricate MPS at the micrometer or even at smaller nanometer scales.

Photolithography is a process used to define fine structures by controlling the exposure to UV light. SU‐8 is used as a photoresist for manufacturing MPS molds.^[^ ^69^ ^]^ The photoresist was spin‐coated onto a flat surface such as a silicon wafer and baked to remove the solvent.^[^ ^70^ ^]^ Selective illumination with UV light through a photomask determines the region of crosslinking or dissolution. Then, microstructures can be fabricated on a silicon wafer by removing the uncrosslinked or crosslinked photoresist based on the photoresist type. Compared with 3D‐printed parts, the surface of the fabricated mold had a significantly lower surface roughness.^[^ ^71^ ^]^

Porous membranes with micro‐ or nanoscale pores can be integrated with 3D‐printed components to enhance the structural and functional complexities of MPS platforms. These membranes are fabricated from a variety of materials, which include PDMS, polyethylene terephthalate (PET), and polycarbonate (PC), with material‐dependent fabrication and bonding methods.^[^ ^72^ ^]^ Predefined membrane geometries can be achieved using microstructured channel molds in which PDMS or UV‐curable resins are cast and cured before demolding. This method supports various photopolymers such as polyurethane acrylate, PEGDA, and PDMS.^[^ ^73^ ^]^ Following the oxygen plasma treatment, membranes composed of PDMS, PEGDA, or polyester can be bonded to PDMS substrates.^[^ ^74^ ^]^ In contrast, porous PET and PC membranes are fabricated via track etching, which involves bombarding the membrane surface with high‐energy heavy‐ion beams (several million electron volts) to generate pores ranging in size from nanometers to micrometers.^[^ ^75^ ^]^ These membranes require chemical bonding using agents such as epoxy silane because of their resistance to plasma bonding.^[^ ^76^ ^]^

Electrospinning is another versatile technique for generating nanofibrous membranes. This involves ejecting a polymer solution under a high‐voltage electric field to form nanometer‐scale fibers.^[^ ^77^ ^]^ The resulting fiber morphology (diameter, shape, and alignment) can be tuned by controlling parameters such as the injection rate, applied voltage, and solution viscosity.^[^ ^78^ ^]^ Fibers are collected in a random orientation on a conductive collector; however, alignment can be achieved by modifying the collector geometry. Aligned nanofibers affect cellular orientation and promote parallel cell growth along the fiber axis.^[^ ^79^ ^]^ Electrospun membranes can serve as functional barriers within microchannels. Choi et al. integrated electrospun membranes into DLP‐fabricated microchannels by applying a liquid photopolymer as an adhesive, followed by UV curing.^[^ ^80^ ^]^

Woven‐mesh membranes are frequently employed as porous barriers in MPSs, and they can filter particles across a wide size range (1–1000 µm), with the pore size controlled by the weaving process and thread diameter. Common materials include synthetic polymers such as nylon and PET. Recent advancements in surface modification techniques enabled cell attachment and proliferation on these membranes, expanding their potential utility in biological applications.^[^ ^81^ ^]^

In the following section, we review representative examples of MPS molds and chips fabricated using 3D printing combined with multiscale ancillary techniques.

## 5 MPS molds with multiscale ancillary techniques

The molds and chips fabricated using 3D printing offer advantages over traditional mold‐based replication processes in terms of size and complexity. In addition, 3D‐printed chips demonstrate the potential for integration with various multiscale auxiliary techniques. In this section, we present examples that incorporate ancillary techniques using irreversible bonding and those that do not require bonding.

### 5.1 Assembly with Membrane by Irreversible Bonding

**Figure 6:**
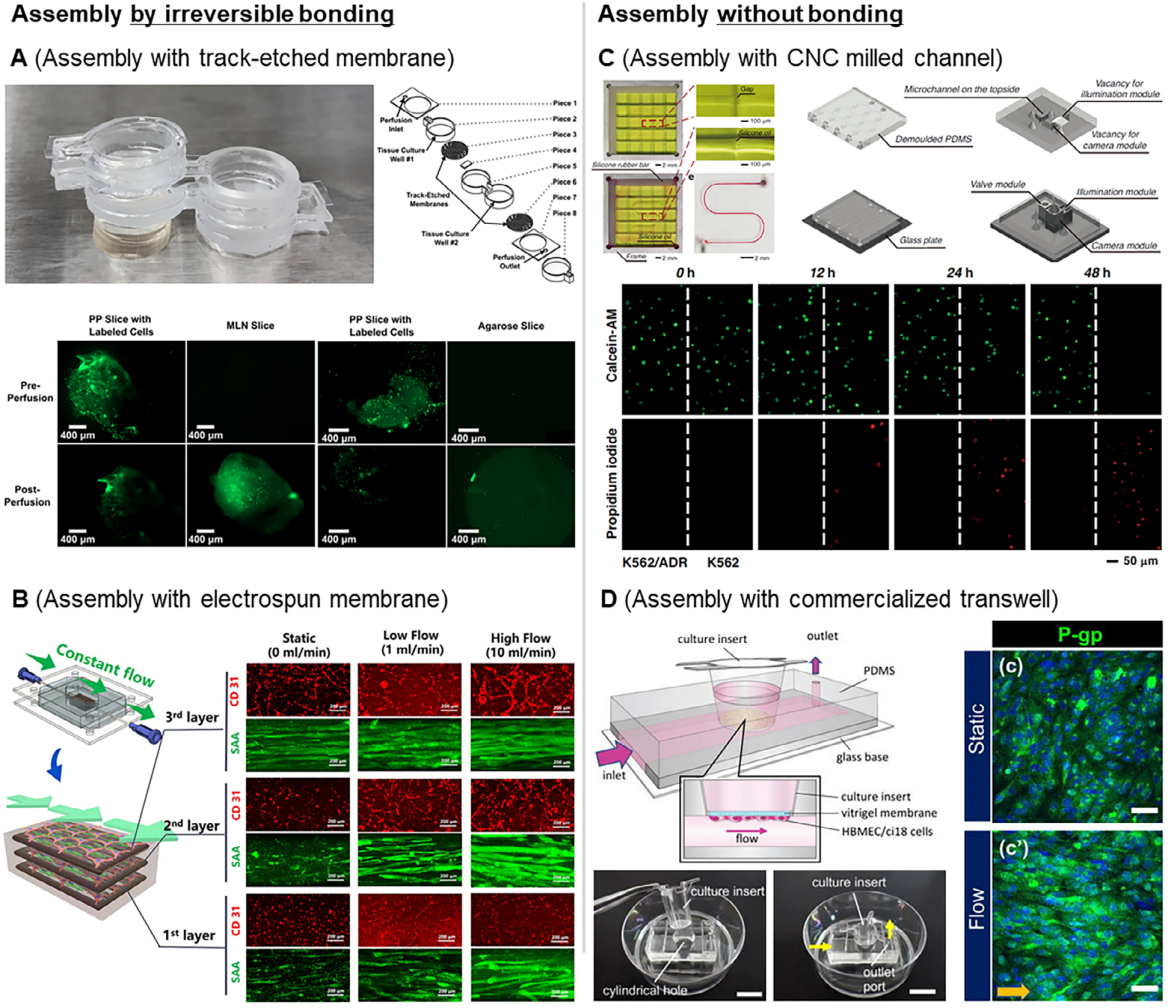
Representative examples of MPS fabricated using multiscale ancillary techniques and 3D‐printed molds. A) This MPS is manufactured through an irreversible bonding process between channels fabricated with a 3D‐printed mold and a track‐etched porous membrane. Direct communication between tissue slices allows integration with electrochemical sensors for the real‐time analysis of inter‐organ molecular exchange. Reproduced from Delong et al. with permission.^[^ ^83^ ^]^ Copyright 2024, Royal Society of Chemistry. B) This MPS is manufactured through an irreversible bonding process between channels fabricated with a 3D‐printed mold and an electrospun membrane. Aligned skeletal muscle tissue assembly enhances myogenic differentiation and induces perfusable vascular‐like structures under stimulation. Reproduced from Wang et al. with permission.^[^ ^88^ ^]^ Copyright 2024, Springer. C) The MPS is constructed by combining CNC‐milled frames and vat‐printed inserts, which enables modular configuration for parallel biological assays, drug screening, and droplet manipulation. Reproduced from Huang et al. under the terms of the CC‐BY‐4.0 license.^[^ ^89^ ^]^ D) The MPS is manufactured by assembling a printed channel with a commercialized Transwell. Fluid flow can be applied to a cell culture using a Transwell through microchannels. Reproduced from Miura et al. under the terms of the CC‐BY‐4.0 license.^[^ ^92^ ^]^

In most cases, planar membranes are assembled by irreversibly bonding them to an MPS chip fabricated using a 3D‐printed mold. Plasma‐activated bonding and crosslinking agents are used for bonding MPS devices to the membranes.^[^ ^82^ ^]^ This irreversible bonding ensured that the assembly remained intact even when mechanical stimulation was applied to the MPS. Delong et al. developed an open‐well multiorgan device incorporating a porous membrane to enable real‐time ex vivo communication between tissue slices. The bidirectional transfer of soluble factors and cells was demonstrated using Peyer's patches and mesenteric lymph nodes, and cytokine diffusion and catecholamine release were monitored (**Figure** **6**A).^[^ ^83^ ^]^ Shrestha et al. demonstrated the hypersecretion of cytokines such as interleukins caused by cigarette smoke extract in a lung‐on‐a‐chip with PC porous membranes.^[^ ^84^ ^]^ Varone et al. observed an increase in surfactant secretion in lung epithelial cells by repeatedly stretching and releasing porous PDMS membranes.^[^ ^85^ ^]^

In these devices, the porous membrane enables interactions between cells on the upper and lower surfaces; however, the cellular environment is often restricted to two dimensions. This restriction can lead to differences in culture conditions, such as cell‐matrix interactions.^[^ ^3^, ^86^ ^]^ A 3D microenvironment can be created using electrospun membranes composed of entangled nanofibers instead of planar membranes to address the heterogeneity of the microenvironment.^[^ ^87^ ^]^ Wang et al. utilized an electrospun poly(ε‐caprolactone) nanofibrous membrane positioned within a microfluidic chip containing parallel microchannels fabricated via 3D printing.^[^ ^88^ ^]^ This electrospun membrane was supported by pillar arrays with diameters and spacings ranging from ≈200 to 600 µm. Skeletal muscle cells cultured on this membrane exhibited an aligned morphology guided by fiber orientation and influenced by fluidic stimulation (Figure 6B).

### 5.2 Assembly Without Irreversible Bonding

In some cases, ancillary parts were assembled without irreversible bonding to the 3D‐printed MPS chip. The absence of irreversible bonding enables the disassembly of parts made with ancillary techniques and 3D‐printed components, thereby enabling the precise analysis of cells cultured within the MPS chip and the potential reuse of these parts. Huang et al. developed a microfluidic mold by integrating CNC‐milled alignment frames with trellis structures. They constructed a versatile and reconfigurable PDMS microfluidic system that can support various biological and chemical assays by assembling these components within a standardized 5 × 5 mm^2^ modular grid (Figure 6C).^[^ ^89^ ^]^

Furthermore, MPS molds can be used for commercially available products. Morimoto et al. proposed an open microfluidic device that utilized a mold produced by vat photopolymerization.^[^ ^90^ ^]^ This device has a cylindrical hole designed to fit a commercially available Transwell. When a Transwell is installed in this hole, its membrane serves as the top surface of the microchannel, enabling fluid flow to be applied to the bottom of the Transwell. In addition, collagen vitrigel membranes, composed of highly dense collagen fibers, can be used instead of porous membranes to create a 3D microenvironment for cells.^[^ ^91^ ^]^ Fluidic shear stress was applied during the experiments, and the culture insert was disassembled from the microfluidic device for biological analysis. Similarly, Miura et al. developed a blood–brain barrier (BBB) model using an MPS chip.^[^ ^92^ ^]^ The BBB model incorporating a nitrogen membrane demonstrated relatively higher P‐glycoprotein activity under fluidic shear stress than under static culture conditions (Figure 6D).

## 6 MPS Chips with Multiscale Ancillary Techniques

The use of augmented 3D‐printing techniques enables the fabricating MPS chips with complex geometries, which enables them to perform functions that are not possible with existing MPS. Moreover, the combination of 3D‐printed MPS chips with various multiscale ancillary techniques expanded the range of the potential applications of MPS devices.

### 6.1 Membrane‐Assembled Chips for Use with Standard Cell Culture Wells and Various Sensors

In vitro cell culture is conventionally performed using standard cell culture plates, and a variety of accessories, including multipipettes and microscopes, are designed to fit these standard cell culture systems. Consequently, MPS chips compatible with existing standardized devices and sensors are being developed for standardizing the entire process. Transwell, a standardized cell culture device comprising an insert and porous membrane that can be mounted on a multiwell plate, is a representative co‐culture system. The porous membrane enabled the transfer of chemokines between cells cultured on both the upper and lower surfaces of the membrane.^[^ ^93^ ^]^

**Figure 7:**
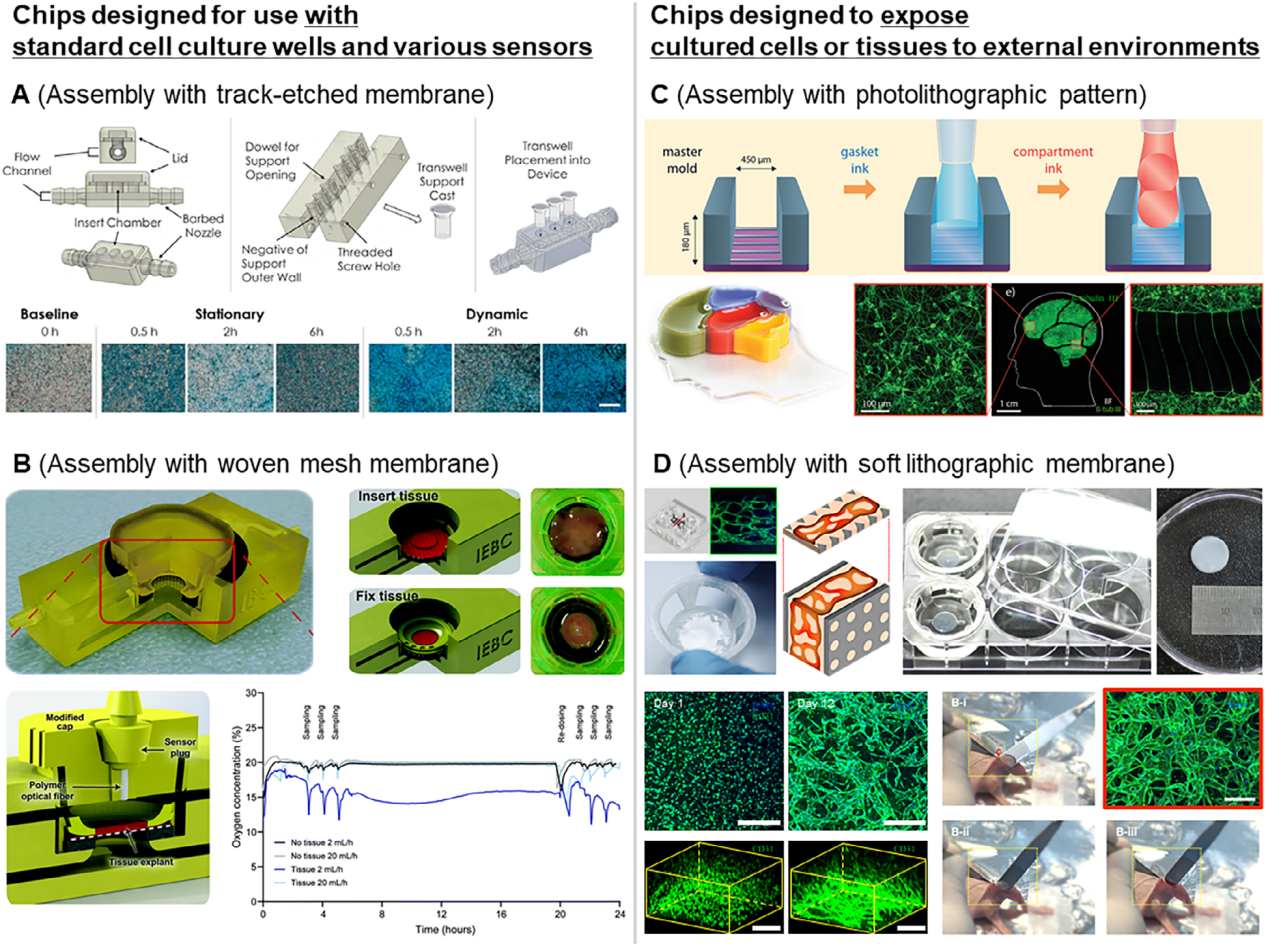
Representative examples of 3D‐printed MPS chips with multiscale ancillary techniques. A) The MPS is manufactured by assembling a track‐etched porous membrane with a channel structure that can be installed on a well plate. The porous membrane is detached easily, which makes analysis easier. Multiple units can be connected through the tubing. Reproduced from Brooks et al. with permission.^[^ ^139^ ^]^ Copyright 2022, KLUWER ACADEMIC PUBLISHERS. B) The MPS is constructed by securing woven mesh membranes in microchannel structures. A woven mesh is used for capturing the extracted tissue and delivering the drug to thattissue. The oxygen consumption of the extracted tissue can be determined in real time by connecting an optical fiber to the upper surface of the microchannel structure. Reproduced from Amirabadi et al. under the terms of the CC‐BY‐NC‐3.0 license.^[^ ^96^ ^]^ C) MPSs are manufactured by 3D printing on silicon wafers with a photolithographic pattern. Open‐well neural chips were produced using 3D printing for macro parts and photolithography for elaborate parts such as axon channels with several micrometers. Reproduced from Kajtez et al. under the terms of the CC‐BY‐4.0 license.^[^ ^98^ ^]^ D) This MPS is produced by scaling up the capillary network MPS. Micropost arrays of the capillary network MPS are replaced with porous soft lithography membranes assembled using 3D‐printed Transwell‐like structures. The therapeutic effect of MPS on ischemia is demonstrated by transplanting the capillary network tissue construct cultured in the scaled‐up MPS. Reproduced from Bang et al. with permission.^[^ ^99^ ^]^ Copyright 2022, WILEY.

Dogan et al. developed a stackable Trans well structure using vat photopolymerization.^[^ ^94^ ^]^ This stackable design enables multiple membranes to be positioned at optimized distances within a single well, facilitating the co‐culture of different cell types. This approach addresses the limitations of the existing Transwell system, which only permits the coculture of cells attached to the upper and lower surfaces of a single membrane. In addition, the traditional Transwell system lacks channels, preventing the application of flow to the cells. Brooks et al. used a vat photopolymerization process to overcome this limitation and fabricate an MPS with a form similar to that of a Transwell, but with embedded microchannels.^[^ ^139^ ^]^ These microchannels enable the precise control of the flow and direction of the upper surface of the porous membrane, which can be easily detached for imaging and analysis. In addition, this MPS can be mounted on a multielectrode array, which is a sensor system that analyzes neuronal signals, facilitating studies on the interaction between blood vessels and neurons (**Figure** **7**A).

Amirabadi et al. proposed a device fabricated by vat photopolymerization for the ex vivo culture of tissue explants.^[^ ^96^ ^]^ This chip was designed to hold the tissue explant on a porous membrane, thereby enabling the upper and lower surfaces of the tissue to be in contact with independent microchannels. This configuration enables separate flow supply to the upper and lower surfaces of the tissue. In addition, an oxygen concentration sensor integrated into the microchannel enabled the measurement of oxygen consumption during the ex vivo tissue culture (Figure 7B).

### 6.2 Multiscale Ancillary Techniques‐Assembled Chips for Exposing Cultured Cells/Tissues To External Environments

In conventional MPS devices, microchannels are formed via photolithography and PDMS replica molding, which results in closed channels. This design limits access to the microchannels and restricts in‐depth analysis.^[^ ^57^, ^97^ ^]^ In contrast, 3D‐printed MPS devices replicate in vivo conditions at the centimeter scale and offer improved compatibility with diverse analytical tools through a multiscale ancillary technique. Kajtez et al. fabricated a PDMS wall with a high aspect ratio using a PDMS extrusion method.^[^ ^98^ ^]^ The resulting MPS achieved axonal isolation and directional axon growth through geometrical confinement (termed an axon diode) while exposing the soma of the neuron to an open compartment. This open‐compartment MPS not only reduced neuronal necrosis within the chip but also enabled electrophysiological analysis using patch clamping (Figure 7C).

In photolithography‐based fabrication, the size of the engineered tissues is limited to the micrometer scale. Scaling up MPS constructs is essential to utilize laboratory‐grown tissues for regenerative medicine. Inspired by the concept of MPSs, Bang et al. developed a scaled‐up vascularized tissue construct with self‐assembled vascular networks within a hydrogel. This was achieved by preparing a structure that encased an endothelial cell‐laden hydrogel in a disk shape using two porous membranes attached to a 3D‐printed Transwell‐like frame.^[^ ^99^ ^]^ This chip has a capillary network tissue construct with a diameter of more than 10 mm and a thickness exceeding 1 mm. The porous membranes were made of PEGDA, which allowed them to spontaneously detach from the frame and enable the withdrawal of vascularized tissue constructs without causing damage. Furthermore, they demonstrated the superior regenerative capability of the vascularized tissue construct when transplanted into a hindlimb ischemia animal model, compared to cases using cell‐only, hydrogel‐only, and cell‐gel mixtures (Figure 7D). The details of the MPS devices using a combination of 3D‐printing and multiscale ancillary techniques with a focus on their features are summarized in **Tables** **3** and **4**.

**Table 3:** 3D‐printed MPS molds/chips with multiscale ancillary techniques.

Refs.	3D‐printing method	Ancillary techniques	Tissue or organ type	Cell types	Cell culture environment
	Device features				
	Results				
3D‐printed MPS molds with multiscale ancillary techniques – With irreversible bonding					
Delong et al.^[^ ^83^ ^]^	Vat photopolymerization	Track‐etched membrane (Polycarbonate)	Lymph node tissue	Mesenteric lymph node slices	2D (On the membrane surface)
	Fabrication of an open‐well chip with detachable tissue compartments				
	Observing real‐time cytokine and molecular exchange between tissue slices				
Shrestha et al.^[^ ^84^ ^]^	Vat photopolymerization	Track‐etched membrane (Polycarbonate)	Lung tissue	Calu‐3 (Lung cancer cells)	2D (On the membrane surface)
	Easy access for cigarette smoke to lung tissue by the upper channel of the membrane opened to the outside				
	Hypersecretion of cytokines from lung tissue after cigarette smoking				
Varone et al.^[^ ^85^ ^]^	Vat photopolymerization	Soft lithographic membrane (PDMS)	Vascularized lung tissue	Primary alveolar epithelial cells Lung fibroblast Lung microvascular endothelial cells	2D (On the membrane surface)
	Repeated stretching and releasing of chip				
	Increased surfactant secretion in alveolar epithelial cells by repeated stretching and releasing				
Wang et al.^[^ ^88^ ^]^	Extrusion	Electrospun membrane (Poly(caprolactone))	Skeletal muscle tissue	C2C12 (Skeletal muscle cells)	2.5D (On a nanofibrous membrane surface)
	Constructing modular nanofibrous scaffolds to replicate the aligned architecture of skeletal muscle tissue				
	Supporting myotube alignment, myogenic differentiation, and perfusion‐induced vascular‐like network formation				
3D‐printed MPS molds with multiscale ancillary techniques–Without irreversible bonding					
Huang et al.^[^ ^89^ ^]^	Vat photopolymerization	CNC milled mold	Hematologic cancer Airway epithelium tissue	Myelogenous leukemia cells Adriamycin‐resistant leukemia cells Bronchial epithelial cells	2D (On the surface of the 3D‐printed part)
	Modular assembly of PDMS microfluidic chips				
	Reduced viability in drug‐sensitive leukemia cells				
Morimoto et al.^[^ ^90^ ^]^	Vat photopolymerization	Transwell (Commercially available)	Vascularized muscle tissue	C2C12 (Myoblasts) HUVECs	2D (On the vitrigel surface)
	Microchannel structure for flow under the Transwell				
	Aligned endothelial cells in the flow direction and myoblasts perpendicular				
Miura et al. ^[^ ^92^ ^]^	Vat photopolymerization	Transwell (Commercially available)	Blood–brain barrier	Brain microvascular endothelial cells	2D (On the vitrigel surface)
	Microchannel structure for flow under the Transwell				
	Increased P‐glycoprotein activity in the blood–brain barrier under flow condition				
3D‐printed MPS chips with multiscale ancillary techniques – Without irreversible bonding					
Rauti et al.^[^ ^95^ ^]^	Vat photopolymerization	Track‐etched membrane (PC)	Vasculature	HUVECs	2D (On the membrane surface)
	Microchannel structure to apply flow to the upper surface of the Transwell, where each module can be connected through tubing				
	Decreased permeability of vasculature under flow condition				
Amirabadi et al.^[^ ^96^ ^]^	Vat photopolymerization	Woven mesh membrane	Intestinal colon tissue	Excised intestinal colon tissue	3D (Excised intestinal colon tissue)
	Microchannel structure to connect the sensor plug to measure the oxygen concentration				
	Measurement of oxygen consumption by the extracted tissue at various flow rates in the microchannel				
3D‐printed MPS chips with multiscale ancillary techniques – Without irreversible bonding					
Kajtez et al.^[^ ^98^ ^]^	Extrusion	Photolithography	Neural circuit	Neural stem cells	2D (On glass)
	Fabrication of a microfluidic neural circuit with an open compartment by extruding the partition wall on the pattern of the mold made by photolithography				
	Analysis of neuronal activity through patch clamp and reduction in neuronal necrosis				
Bang et al.^[^ ^99^ ^]^	Vat photopolymerization	Soft lithographic membrane (PEGDA)	Microvasculature	HUVECs Lung fibroblasts	3D (In hydrogel)
	Microstructure for scaling up tissue				
	Centimeter‐sized vascularized tissue for treating ischemia				

**Table 4:** Emerging 3D‐printing‐related techniques for MPS development.

Technique	Technological readiness level [TRL]	Integration feasibility with MPS	Potential impact on the MPS field	Key strengths	Limitations
Melt electrospinning	Medium (4–5)	Moderate	Scaffold microstructure	High‐resolution	Slow throughput
			ECM mimicry	Biofabrication	Channel structure
TPP	Low–medium (3–4)	Moderate–Low	Submicron precision	Ultrafine 3D patterning	Small build volume
				High resolution	Specialized setup
CAL	Low (2–3)	Low	Rapid volumetric printing	Fast fabrication	Low accessibility
				Support‐free	
4D printing	Very low (1–2)	Low	Dynamic microenvironment modulation	Shape change	Conceptual stage

## 7 Perspective

**Figure 8:**
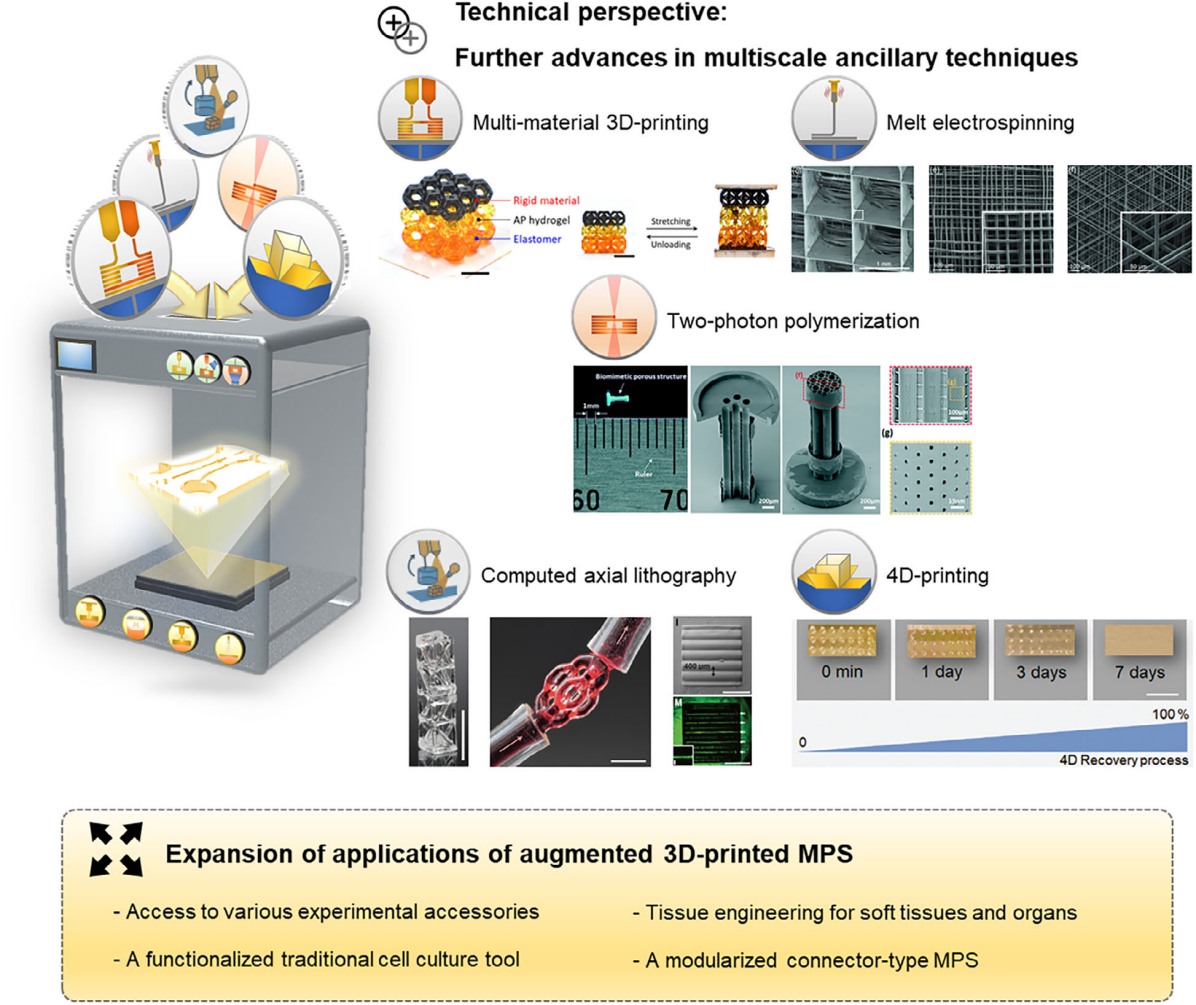
Perspective of augmented 3D‐printed MPS. Multiscale ancillary techniques may be further integrated to develop more advanced 3D‐printed MPS. Representative multiscale ancillary techniques that may be further introduced include multimaterial 3D printing, melt electrospinning, two‐photon polymerization, computed axial lithography, and 4D printing. (Multimaterial 3D printing–Reproduced from Ge et al. under the terms of the CC‐BY‐NC‐4.0 license.^[^ ^101a^ ^]^ Melt electrospinning – Reproduced from Brown et al. with permission.^[^ ^107b^ ^]^ Copyright 2011, WILEY; Two‐photon polymerization–Reproduced from Wei et al. with permission.^[^ ^110c^ ^]^ Copyright 2022, ROYAL SOCIETY OF CHEMISTRY; Computed axial lithography‐Reproduced from Toombs et al. with permission.^[^ ^118^ ^]^ Copyright 2022, AMERICAN ASSOCIATION FOR THE ADVANCEMENT OF SCIENCE; and 4D printing–Reproduced from Miao et al. under the terms of the CC‐BY‐4.0 license).^[^ ^123b^ ^]^ Physiology and pathology modeling for drug screening is the main use for the existing MPS. The use of augmented 3D‐printed MPS is expected to go beyond the primary use of existing MPS and be applied to a wide variety of fields.

Numerous MPS devices employing 3D‐printing techniques and various multiscale ancillary techniques are currently under development. Despite the advancements achieved with these augmented 3D‐printed MPS devices, there is still significant potential for technological progress. **Figure** **8** illustrates the remaining challenges and opportunities to future development, while Table 4 summerizes representative technological strategies and current advancements.

## 8 Introduction of Advanced Manufacturing Techniques

### 8.1 Multimaterial 3D Printing

Multimaterial 3D‐printing technology can be used for extrusion, photopolymer jetting, and vat photopolymerization. In multimaterial 3D‐printing processes, both extrusion and photopolymerization utilize dual nozzles,^[^ ^31^, ^100^ ^]^ whereas vat photopolymerization is achieved by changing the resin bath during printing.^[^ ^101^ ^]^ In addition, a hybrid method that alternates between photopolymer jetting and vat photopolymerization has been developed.^[^ ^102^ ^]^

Multimaterial 3D printing presents structural complexities. For example, a device element can be selectively dissolved by incorporating a water‐soluble material into a frame made of an insoluble material.^[^ ^100^ ^]^ Stretchable devices can be created in a particular area by patterning rigid and flexible parts together into a single piece.^[^ ^31^, ^101^ ^]^ In addition, a porous membrane can be replaced by printing a low‐permeability part directly into the device,^[^ ^101b^ ^]^ and devices with integrated sensors can be produced by printing electrodes during fabrication.^[^ ^102^ ^]^

### 8.2 State‐of‐the‐Art Electrospinning

A method for decorating the surfaces of 3D‐printed parts with electrospun nanofibers was recently developed.^[^ ^103^ ^]^ This method involves immersing the 3D‐printed part in a solution containing melted electrospun nanofiber segments, which results in a nanotopography on the surface of the printed part. Further, another technique was developed to fill empty spaces within 3D‐printed parts with electrospun nanofibrous scaffolds.^[^ ^104^ ^]^ In this technique, a solution of electrospun nanofiber segments is infused into the vacant space of a 3D‐printed part, followed by lyophilization. Thus, electrospun nanofibers have the potential to replace porous membranes and serve as hydrogel‐like niches for cell cultures.

Thus far, all electrospinning methods described in this paper use a polymer dissolved in a solvent, which is a process commonly referred to as solution electrospinning. However, there has been growing interest in electrospinning using melted polymers.^[^ ^105^ ^]^ Melted electrospinning does not require a solvent, which helps completely avoid solvent‐associated toxicity and is advantageous for fabricating MPS that provide a microcellular environment.^[^ ^106^ ^]^ Melt electrospinning writing (MEW), which is a subset of extrusion‐based 3D‐printing techniques, is used to fabricate highly reproducible and controlled structures through melt electrospinning. During the MEW process, parameters such as the viscosity and charge of the molten polymer, mass flow rate, collector speed, and electric field must be precisely controlled.^[^ ^107^ ^]^ Microfluidic devices using MEW have been proposed^[^ ^108^ ^]^ and MEW is expected to play a significant role in the development of future MPS chips.

### 8.3 Two‐Photon Polymerization

Two‐photon polymerization (TPP) is a technology used to fabricate a 3D structure using two‐photon absorption (TPA). When a two‐photon laser is focused on a photopolymer resin, the resin is cured by absorbing the laser energy at the focal point; this process is referred to as TPA. The TPP process can produce 3D structures with nanometer‐scale precision smaller than the laser wavelength and can be categorized as a form of photopolymerization.^[^ ^109^ ^]^

Recently, numerous studies have explored the use of TPP to create intricate cell‐scaffold structures. TPP can be employed to engineer a neural circuit scaffold that directs nerve cell growth in specific directions^[^ ^110^ ^]^ and can be used to build artificial blood vessels in the form of tubes with nanopores.^[^ ^111^ ^]^ The TPP technology can be integrated with other technologies to create multiscale structures. For example, a porous membrane chip can be fabricated without a mold by first creating a microchannel chip using vat photopolymerization, followed by creating a porous membrane within the microchannel using TPP.^[^ ^112^ ^]^ Similarly, a nanomicrochannel structure can be created by fabricating a microchannel mold on a wafer using photolithography and a nanochannel mold using the TPP process.^[^ ^113^ ^]^ In addition, integrated nano‐/micro‐centimeter channel chips have been developed by combining nano/microchannel fabrication via TPP and photolithography with a centimeter‐scale channel fabricated by CNC milling.^[^ ^114^ ^]^

Two‐photon lasers can ablate materials precisely by focusing on high‐energy levels at a specific point.^[^ ^115^ ^]^ This capability enables the fabrication of multiscale chips by ablating microchannels with a two‐photon laser after producing millimeter‐scale channels through vat photopolymerization.^[^ ^116^ ^]^

### 8.4 Computed Axial Lithography

Computed axial lithography (CAL) is a promising approach for the rapid and precise fabrication of microfluidic devices.^[^ ^117^ ^]^ Unlike conventional additive manufacturing techniques that rely on layer‐by‐layer deposition, CAL achieves volumetric 3D printing by projecting computed light fields onto a rotating vat of photosensitivity.^[^ ^118^ ^]^ Through inverse tomographic reconstruction, a series of 2D light patterns is generated and projected from multiple angles, enabling selective photopolymerization of complex 3D geometries within seconds.^[^ ^119^ ^]^ This single‐step, support‐free process produces parts with smooth surfaces and eliminates the need for post‐processing to remove layering artifacts, which is a significant advantage in microfluidics, where surface roughness and dimensional fidelity directly affect fluid flow and device performance.

CAL could be particularly useful for developing organ‐on‐a‐chip systems, vascularized tissue scaffolds, and multiplex analysis platforms.^[^ ^120^ ^]^ Further, microenvironments suitable for cell culture, drug screening, and disease modeling could be fabricated by integrating CAL with biocompatible or functional photopolymers. Ongoing advancements in resolution, resin chemistry, and computational reconstruction algorithms are expected to further expand the applicability of this technology to microscale engineering.

### 8.5 4D Printing

Temporal control is essential for accurately replicating an in vivo environment. 4D printing offers a solution to this problem. 4D printing can be used to create structures that can change their shapes and functions in response to various stimuli and conditions.^[^ ^121^ ^]^ A range of 3D‐printing techniques including MEW and TPP can be employed to produce structures that morph into various shapes.^[^ ^122^ ^]^ Shape morphing can be achieved using various elements, including shape memory polymers,^[^ ^123^ ^]^ patterned structures,^[^ ^122^, ^123^ ^]^ structures with multiple thicknesses per unit area,^[^ ^122b^ ^]^ structures containing materials with diverse properties,^[^ ^124^ ^]^ and structures incorporating magnetic nanoparticles.^[^ ^125^ ^]^ 4D printing has been recently used in cellular biology studies. One study demonstrated that the differentiation of neural stem cells was enhanced in shape‐memory polymer microwell arrays transformed into a series of micropatterns over time.^[^ ^123b^ ^]^ Another study used shape‐memory polymers to produce vascular grafts with small diameters by morphing tubular polymers into planar shapes, attaching endothelial cells to their surfaces, and restoring the polymers to their original forms.^[^ ^126^ ^]^ Integrating 4D printing with the MPS technology can provide an even more refined tissue simulation model.

#### 8.5.1 Expanding the Applications of Augmented 3D‐Printed Multiscale MPS

MPS is used for drug screening, and it predicts drug responses more accurately than that with conventional 2D cell cultures and animal models.^[^ ^127^ ^]^ This advantage can be attributed to the MPS being able to more closely mimic human physiology.^[^ ^128^ ^]^ The recent advancements in augmented 3D printing and the development of multiscale MPS have enabled applications that were previously unachievable using conventional MPS technologies.

Conventional MPS technologies suffer from limitations in integrating external components such as holders, connectors, light‐emitting diodes, sensors, and filters. Augmented 3D printing addresses this issue by enabling the fabrication of MPS with built‐in features tailored to mount such accessories.^[^ ^129^ ^]^ Further, MPS can be fabricated with threaded ports to ensure compatibility with general‐purpose laboratory components.^[^ ^130^ ^]^

Microfluidic chips with internal microchannels and integrated connector structures have also been developed using 3D printing.^[^ ^131^ ^]^ These modular systems eliminate the need for external tubing, which helps adsorb drugs and provide accurate dosing.^[^ ^132^ ^]^ Unlike conventional human‐on‐a‐chip systems that require permanent connectivity throughout the experiment, the connector‐based MPS architectures enable the flexible assembly and disassembly of individual modules.

In addition, augmented 3D printing expands the utility of standard cell culture tools such as well plates. Although 3D‐printed MPS in well plate formats have been discussed in this review,^[^ ^19a,b^ ^]^ earlier versions lacked perfused channels between wells. New designs with perfusion‐enabled lids or integrated channels have been proposed to overcome this problem.^[^ ^133^ ^]^ Augmented 3D printing, with its ability to generate multiscale and geometrically complex structures, supports the development of highly functionalized MPS for advanced cell culture applications.

Augmented 3D‐printed MPS also presents new opportunities for tissue engineering. The existing advantages of MPS, such as vascularization and spatial organization of organoids, are enhanced in augmented systems.^[^ ^45^, ^99^ ^]^ These technologies are expected to considerably improve the fabrication of vascularized tissues and organ models by more faithfully replicating the physiological features of human organs. Given the current advancements in organoid development, integrating organoid technologies with augmented MPS can further enhance in vivo‐like performance.^[^ ^134^ ^]^ Mesh‐based printing formats support the construction of tissues with curved surfaces.^[^ ^135^ ^]^ These advances have augmented 3D‐printed MPS as powerful platforms for next‐generation tissue engineering and regenerative medicine.

## 9 Conclusion

MPS technology was developed for drug screening because it can accurately recapitulate the physiology and pathophysiology of human organs. However, existing MPS manufacturing methods face challenges when fabricating complex 3D structures. To address this issue, 3D‐printing technology was introduced to produce MPS with intricate 3D configurations. Despite these advancements, 3D‐printing techniques for MPS fabrication are suitable only for structures ranging from several hundred micrometers to a few centimeters. Therefore, combining other technologies tailored to specific scales is essential for producing multiscale MPS ranging from hundreds of micrometers to tens of centimeters. Multiscale MPS fabrication relies on diverse ancillary techniques, including CNC milling, photolithography, track etching, and electrospinning.

We introduced the concept of an augmented 3D‐printed MPS that merges 3D‐printing methods with these multiscale ancillary techniques. This augmented approach enables creating highly sophisticated structures and facilitates various functionalities beyond the capabilities of existing MPS. In the future, we expect that more advanced multiscale ancillary techniques will be integrated into augmented 3D‐printed MPS, and the application scope of these advanced systems will expand beyond drug screening, thus opening new opportunities in areas such as regenerative medicine, disease modeling, and personalized medicine.

## Conflict of Interest

The authors declare no conflict of interest.
